# Development and testing of an opioid tapering self-management intervention for chronic pain: I-WOTCH

**DOI:** 10.1136/bmjopen-2021-053725

**Published:** 2022-03-16

**Authors:** Harbinder Kaur Sandhu, Jane Shaw, Dawn Carnes, Andrea D Furlan, Colin Tysall, Henry Adjei, Chockalingam Muthiah, Jennifer Noyes, Nicole K Y Tang, Stephanie JC Taylor, Martin Underwood, Adrian Willis, Sam Eldabe, the I-WOTCH Team, Sharisse Alleyne

**Affiliations:** 1 Warwick Clinical Trials Unit, Warwick Medical School, University of Warwick, Coventry, UK; 2 Department of Pain Medicine, James Cook University Hospital, Middlesbrough, UK; 3 Wolfson Institute of Population Health, Barts and The London School of Medicine and Dentistry, Queen Mary University of London, London, UK; 4 Toronto Rehabilitation Institute, University Health Network, Department of Medicine, University of Toronto, Toronto, Ontario, Canada; 5 Department of Medicine, Institute for Work & Health, Toronto, Ontario, Canada; 6 UNTRAP, University of Warwick, Coventry, West Midlands, UK; 7 SUCE, Coventry University, Coventry, West Midlands, UK; 8 Department of Psychology, University of Warwick, Coventry, West Midlands, UK; 9 University Hospitals Coventry and Warwickshire, Coventry, UK

**Keywords:** pain management, rehabilitation medicine, medical education & training, pain management, primary care, clinical trials

## Abstract

**Objectives:**

To describe the design, development and pilot of a multicomponent intervention aimed at supporting withdrawal of opioids for people with chronic non-malignant pain for future evaluation in the Improving the Wellbeing of people with Opioid Treated CHronic pain (I-WOTCH) randomised controlled trial.

**Design:**

The I-WOTCH intervention draws on previous literature and collaboration with stakeholders (patient and public involvement). Intervention mapping and development activities of Behaviour Change Taxonomy are described.

**Setting:**

The intervention development was conducted by a multidisciplinary team with clinical, academic and service user perspectives. The team had expertise in the development and testing of complex health behaviour interventions, opioid tapering and pain management in primary and secondary care, I.T programming, and software development—to develop an opioid tapering App.

**Participants:**

The I-WOTCH trial participants are adults (18 years and over) with chronic non-malignant pain using strong opioids for at least 3 months and on most days in the preceding month.

**Outcomes:**

A multicomponent self-management support package to help people using opioids for chronic non-malignant pain reduce opioid use.

**Interventions and results:**

Receiving information on the impact of long-term opioid use, and potential adverse effects were highlighted as important facilitators in making the decision to reduce opioids. Case studies of those who have successfully stopped taking opioids were also favoured as a facilitator to reduce opioid use. Barriers included the need for a ‘trade-off to fill the deficit of the effect of the drug’. The final I-WOTCH intervention consists of an 8–10 week programme incorporating: education; problem-solving; motivation; group and one to one tailored planning; reflection and monitoring. A detailed facilitator manual was developed to promote consistent delivery of the intervention across the UK.

**Conclusions:**

We describe the development of an opioid reduction intervention package suitable for testing in the I-WOTCH randomised controlled trial.

**Trial registration number:**

ISRCTN49470934.

**Strengths and limitations of this study**

- The Improving the Wellbeing of people with Opioid Treated CHronic pain (I-WOTCH) Intervention draws on psychological and behaviour change frameworks.
- The I-WOTCH intervention was developed with key stakeholders including patient and public involvement (those with chronic-non-malignant pain and experience of opioid use and/or tapering).
- The pilot phases and feasibility testing gave valuable feedback and changes were made to the intervention accordingly.
- At the time of designing the intervention, there was limited previous work and information to inform content of the intervention.

## Introduction

Pain, and pain related disorders, continue to be the leading cause of disability and disease burden globally,^1^ with low back pain making the largest contribution to years lived with disability. In England, at least 8 million people (15% of the population) have moderate-to-severe persistent (chronic) pain^2^ defined as *pain that lasts or recurs for more than 3 months*.^3^ Over the past few decades, there has been a global epidemic of opioid prescribing for chronic non-malignant pain. A 2020 systematic review found that 30% of people with chronic non-malignant pain are prescribed opioid medication and, globally, this has steadily increased until recently with time.^4^ In the UK, prescribing rates have decreased slightly over recent years; however, the number of prescriptions remains high.^5^ Long-term use of opioids leads to tolerance and loss of effective pain relief. Adverse consequences include opioid-induced hyperalgesia, endocrine hypogonadism, drowsiness, a high risk of dependency, opioid use disorder, sleep apnoea, immune suppression, falls leading to increased fractures (particularly a risk in the elderly population) and increased risk for overdose and death.^6^ There are limited strategies to help with risk mitigation and evidence based interventions to help people with chronic non-malignant pain withdraw from opioids.^7^ A 2020 systematic review found ten randomised controlled trials (n=835) of patient-focused opioid de-prescribing interventions targeting people with chronic non-malignant pain. These included: dose reduction protocols (weekly reduction of 10%); opioid replacement including (buprenorphine, morphine sulphate or oxycodone hydrochloride or varenicline; non-pharmacological therapies including mindfulness (vs active control or support group); therapeutic interactive voice response programme (vs usual care); meditation; cognitive–behavioural therapy (vs usual care); and electroacupuncture (vs sham). The primary outcome was mean reduction of daily dose in morphine milligram equivalents (MME). Only one study reported a statistically significant difference in the daily dose between groups in favour of the intervention (a study using a dose tapering protocol) (mean difference −27.9 MME/day, 95% CI −41.1 to −14.7).^8^ None of these interventions reported increases in opioid cessation in the intervention groups. Overall, the authors were unable to recommend any particular deprescribing strategy due to the small number of studies and heterogeneity of the data.^4^

Current recommendations on opioid tapering are based on the best practice and guidelines which need to be supported by further evidence.^9^ Here, we describe the development of a multicomponent opioid tapering programme (incorporating group and one to one sessions) as part of the I-WOTCH study (Improving the Wellbeing of people with Opioid Treated CHronic pain), funded by the National Institute of Health Research (14/224/04). The I-WOTCH study protocol has been published previously.^10^

## Methods

The I-WOTCH intervention was developed in collaboration with the target population (those with chronic non-malignant pain and experience of opioid use). It employed theory and evidence-based implementation (with a view to implementation in the real world should it be effective) and included digital technologies to generate opioid tapering plans.^11^ The Medical Research Council Framework^12^ for designing complex interventions, evidence-based interventions^13^ and core theoretical principles were used to inform the design of content, structure and delivery of the intervention.

**Figure 1:**
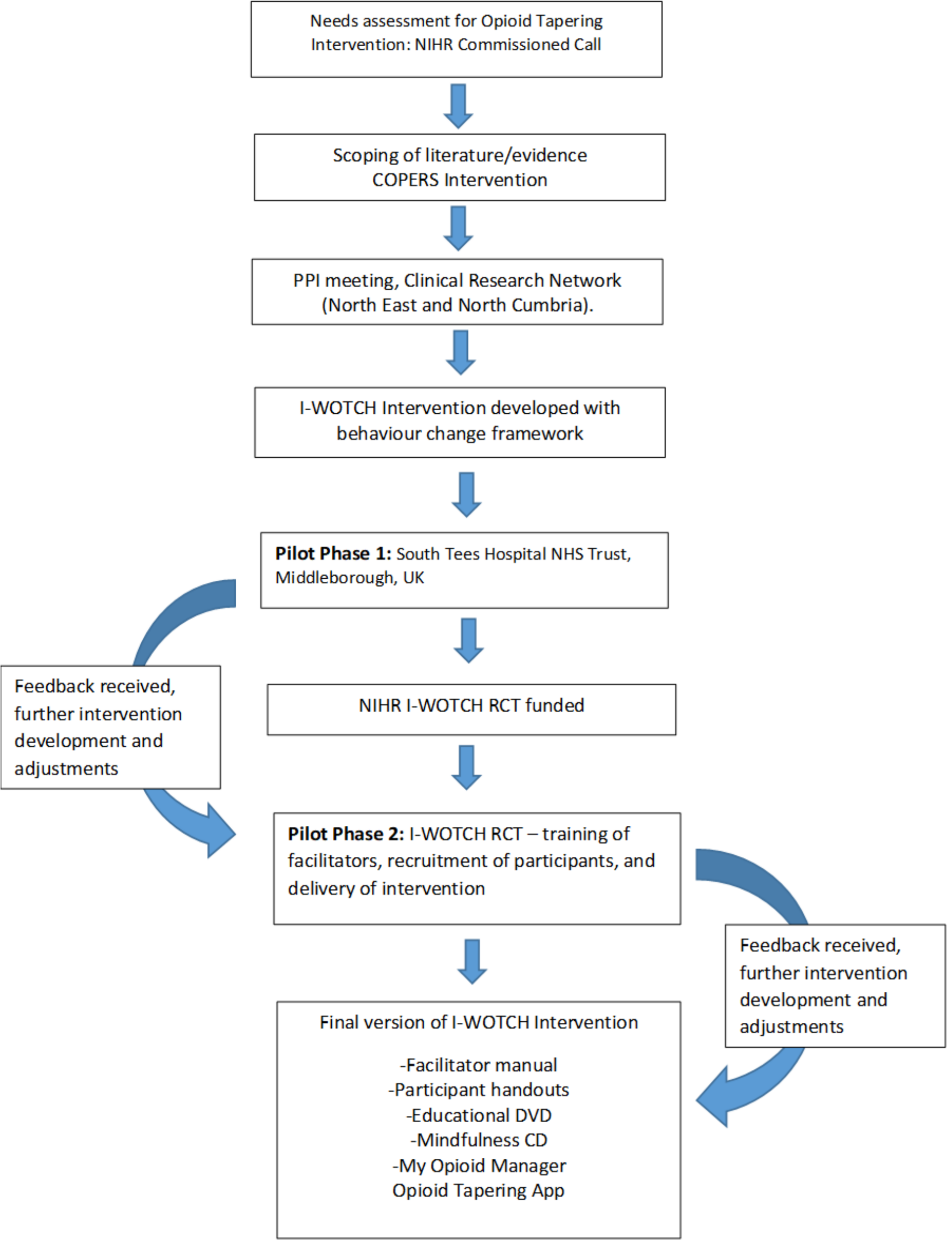
Stages of Improving the Wellbeing of people with Opioid Treated CHronic pain (I-WOTCH) intervention development. NIHR, National Institute for Health Research; RCT, randomised controlled trial.

Key stages of the intervention development are shown in figure 1. Adjustment and adaptation to the intervention were implemented inline with feedback received from stakeholders (service users, clinicians and facilitators delivering the I-WOTCH intervention).

### Aims and objectives of the I-WOTCH intervention

In line with the overall study, the aims of the I-WOTCH intervention were:

1. To reduce opioid and healthcare use for people with chronic, non-malignant pain.
2. To increase study participants’ self-efficacy (confidence) to reduce opioid medication and implement self-management strategies of pain.
3. To improve quality of life and help people live better with pain.

Objectives:

1. To provide education using a range of teaching methods; group discussion, problem-solving, experiential learning and case studies.
2. To provide an environment which enhances motivation to reduce opioid use through group cohesion and one to one support.
3. To provide an overall cost-effective intervention to be implemented in healthcare services.

**Figure 2:**
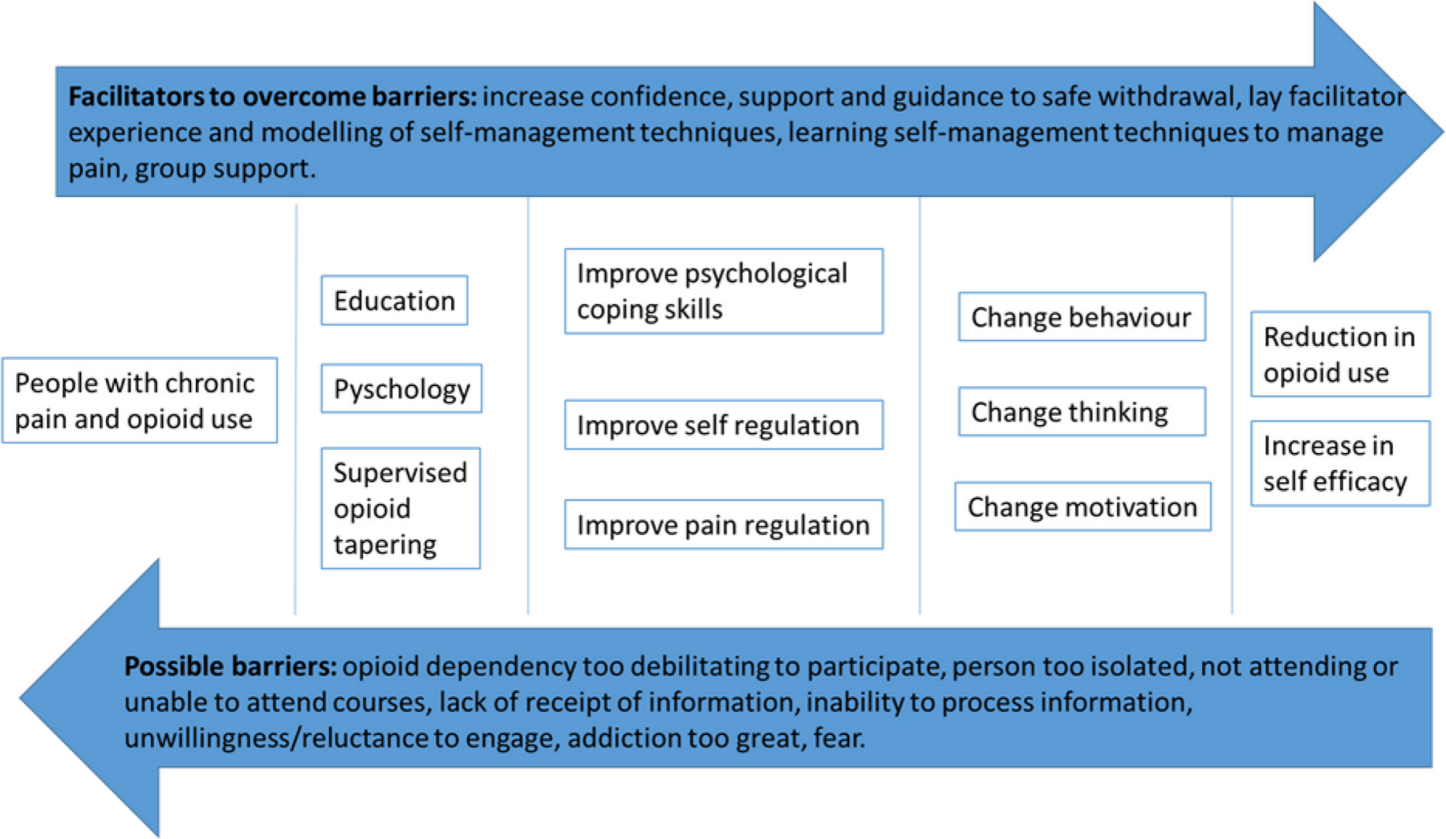
Reducing opioids for people with chronic non-malignant pain.

Defining the aims and objectives enabled us to consider what we wanted to achieve, how and for what purpose. In addition, we were aware of potential facilitators and barriers that could influence engagement with the intervention and the procedures of the trial. Figure 2 shows the direction of travel we were aiming for and what we needed to consider when designing the detail of the intervention and mechanism of behaviour change.

### Patient and public involvement

During the development stages of I-WOTCH, we held two PPI meetings with the Clinical Research Network (North East and Cumbria) at The James Cook University Hospital (South Tees Hospitals NHS Foundation Trust). A total of 19 volunteer participants (people with chronic pain and experience of opioid therapy and/or opioid tapering) attended. Discussions were facilitated by members of the study team (HS, DC, JS and SE) and included, intervention structure and design, content (topics to cover which would potentially increase motivation and confidence to taper opioids), length of programme, where the intervention should be delivered, support during opioid tapering (including frequency of contact with healthcare professionals) and delivery of the intervention (who should deliver the intervention) (table 1). In addition to this, two lay advisors who were part of the I-WOTCH study recruited via Universities/User Teaching^14^ gave considerable input into the design of, and training to deliver, the intervention.

**Table 1:** Feedback from PPI informing intervention development

Discussion topic	Feedback informing intervention development
Behaviour change	Agreed aims should be a reduction in opioid consumption and engagement in the I-WOTCH programme. Behaviour change needs to be accepted before opioid reduction can occur.
Understanding motivation to change behaviour	Changing medication and reducing medication can be motivated by: (i) a trade-off to fill the deficit of the effect of the drug (something else needed that is as effective as the drug they would lose) (ii) reduction in side effects Use of case studies of people who had successfully stopped taking opioids would be useful.
Content and topics to be covered	The intervention would benefit from being informative (opioid education, especially long-term consequences, pros and cons of opioid use and managing withdrawal). The following topics were recommended for inclusion: What is pain Acceptance—pain and learning to live better with pain Impact of pain – and integrate this information with taking medication (Opioids), why and how? The importance of hobbies and having a distraction to manage the pain Offer alternative non-pharmacological ways of coping, for example, mindfulness and relaxation Incorporate movement Guidance on posture and exercise/activity Pacing—not over doing things
Dependency versus addiction	It was felt important to distinguish between dependency and addiction, as some were concerned about the stigma and labels attached to long-term opioid use for chronic pain.
Delivery of I-WOTCH Intervention, who?	Feedback favoured the course to be delivered jointly by a HCP and a lay facilitator (someone who had experience of long-term pain and opioid use/tapering).
Structure of intervention	Group and individual care approaches were valued. Length of the proposed programme (3-day group sessions and ongoing one to one support) was supported. The duration of intervention was not viewed as burdensome given that some had people who had experienced severe withdrawal symptoms, and therefore ongoing support over the 8–10 weeks was needed. There was a consensus that a group-based format and group cohesion would be optimal because of the potential for social comparison, social validation and development of social support. Volunteers identified the impact of opioid use on enhanced day-to-day activities as important evaluation outcomes, including: work productivity, looking after children, and overall functioning.
Communication during study	Volunteers welcomed the idea of having a study website to give participants an opportunity to be updated about the study as a whole and progress.

HCP, healthcare professional; I-WOTCH, Improving the Wellbeing of people with Opioid Treated CHronic pain.

### Opioid tapering and behaviour change

The target behaviour change was defined as the participants engaging in the I-WOTCH intervention: reducing participant opioid use, and implementing non-pharmacological strategies of pain management. The biopsychosocial framework,^15^ Michie’s taxonomy of behaviour change and the COM-B framework for behaviour change (Capability, Opportunity, Motivation) were consulted.^16^ Capability includes psychological capability (eg, can patients engage in the necessary thought processes needed to commit and adhere to the tapering processes?) and physical capability (eg, do participants have the capacity to engage in the tapering?). Psychological capability is broken down to cognitive functioning and executive functioning. To promote cognitive functioning (which includes a range of mental abilities such as learning, problem-solving and attention), we produced handouts of material covered on each day of the programme. This allowed opportunity for participants to recap over the core messages and information in their own time. We also included time for group reflection at the start of each session and summarising discussions at the end of each of the group days (with opportunities for questions). In addition to this, we developed an educational DVD, a mindfulness CD and relaxation CD for each participant (at the time we developed the intervention DVDs and CDs were still in common use). By providing material to take home, we were giving participants an opportunity to revisit and take in the information at their own pace.^17^ Executive functioning includes the capacity to plan and think, explore challenges that may occur (eg, fear of withdrawal symptoms), stay focused on the goal (opioid reduction) and resist temptation.^18^ In the I-WOTCH intervention, we gave participants opportunity to set goals (through an educational session and support in generating goals related to opioid tapering and their general life). We also encouraged self-reflection to identify perceived barriers and facilitators to tapering and gave further guidance to overcome the perceived barriers in the tailored one to one support sessions with the clinical facilitator. Physical capability refers to whether the participants exposed to the I-WOTCH intervention felt they had the right skills to engage in the tapering of their opioids, this may include management of withdrawal, confidence and having structure and support in place. The I-WOTCH intervention was designed to help participants adapt and put into place lifestyle changes.

Opportunity is the second component of the COM-B model. For this, we explored factors external to the individual that would promote opioid tapering. For example, physical opportunity includes costs of opioids and travel, access and availability, developing a tapering plan (clear and informative) and enhancing communication between the clinical facilitator and participant through motivational interviewing (MI) during the tapering processes. In relation to social opportunity, we referred to what other factors may impact the decision to taper such as stigma and cultural beliefs.

Motivation, this refers to both the cognitive motivation and emotional processes to energise and direct the behaviour change of opioid tapering. Reflective processes included exploring perceptions and meaning of chronic pain during the group sessions as well as beliefs about tapering, possible outcomes concerns and self-efficacy. There was opportunity to evaluate and be reflective during the group sessions as well as one to one support. Automatic processes refer to the emotional responses which may occur during the I-WOTCH intervention and these include anxiety, fear, stress and low mood. All topics were covered in the group sessions including recognition of thoughts and emotions and management strategies.

Each component of the I-WOTCH intervention was informed and mapped on to behaviour change taxonomies. The intervention also drew on psychological theories of self-efficacy,^19^ theory of planned behaviour and reasoned action,^20 21^ social learning^22^ and group-based interventions,^23^ cognitive–behavioural change,^24^ MI^25^ and evidence-based interventions for self-managing chronic pain (COPERS)^26^ described in table 2.

**Table 2:** Behaviour change taxonomy and opioid tapering

I-WOTCH group based sessions day 1 (week 1)	Aims	Theoretical underpinnings	Behaviour change taxonomy
Introductions, group work, aims	To allow participants to introduce themselves to the group, encourage participation in a safe and relaxed environment, explore expectations and discuss the I-WOTCH course aims	Social cognitive theory Biopsychosocial theory	Improve bonding and group cohesion. Breaking barriers and encouraging self and social awareness
What causes pain? (pain information)	To increase understanding about long-term pain	Biopsychosocial theory Principles of self-efficacy and acceptance	Credible source
Living with pain (Opioid education I)	To increase understanding about use of opioids for long-term pain and encourage participants to start questioning their own knowledge and beliefs about opioids and why they take them	Biopsychosocial theory Theory of planned behaviour and reasoned action Health beliefs	Information about health consequences
Acceptance	To understand and start to accept pain, with a view to implementing self-management strategies as reduction of opioids occurs	Acceptance and self-management of chronic pain	Goal setting Commitment
Attention control and distraction	To learn how to focus the mind away from pain thoughts and use of opioids	Cognitive–behavioural change Self-management of chronic pain Health beliefs	Distraction
Distraction activity—drawing	An opportunity to practise distraction activity and socially interact with group informally	Cognitive–behavioural change Social learning	Behavioural practice Distraction
Good days, bad days—pain, bearable or not?	To reinforce that pain is not just physiological, it is a psychological, social and an emotional phenomenon	Biopsychosocial theory Health beliefs	Information and antecedents Information about health consequences Reattribution of behaviour
The pain cycle (including opioids) and breaking the pain cycle	To explain and identify unhelpful factors in the pain cycle and learn strategies to break the cycle	Biopsychosocial theory Health beliefs	Behaviour substitution (adding in other behaviours to break cycle)
Posture and movement	To promote body awareness, posture and muscle weakness (managing pain without opioids)	Theory of planned behaviour and reasoned action	Guidelines on exercise, physical therapy principles Mindfulness
Relaxation and breathing	To reduce muscle tension and introduce breathing as a relaxation technique	Cognitive—behavioural change Self-management of chronic pain	Behavioural practice Distraction Body changes
Summary of the day	To consolidate learning of the day and outline aims for group day 2.	Acceptance and principles of self-efficacy	Action planning Verbal persuasion about capability

I-WOTCH group-based Sessions Day 2 (week 2)	Aims	Theoretical underpinnings	Behaviour change taxonomy
Reflections from day 1	To understand and empathise with the group	Social learning Self-efficacy	Improve bonding and group cohesion, social cognitive theory
Stress-busting for Health: Action planning, problem-solving, pacing, SMART goal setting	To help the participants logically and systematically identify problems, free think solutions, set achievable goals and create action plans, as a means of escaping the pain cycle	Cognitive–behavioural change Theory of planned behaviour and reasoned action	Goal setting Comparative imagining of future outcomes Reduce negative emotions Problem-solving
Withdrawal symptoms, case studies (Opioid education II)	To discuss potential withdrawal symptoms that participants might experience if their taper is too quick	Health beliefs Social learning	Social comparison (drawing attention to others’ performance to allow comparison with the person’s own performance) Credible source Comparative imagining of future outcomes
Distraction activity—origami	To learn how to focus the mind away from pain thoughts and use of opioids	Cognitive–behavioural change Social learning	Behavioural practice Distraction
Identifying and overcoming barriers to change	Introduce ideas about unhelpful thoughts, automatic thoughts and errors in thinking. To identify reasons why people stay in the pain cycle, and barriers to change. Introduce positive reframing	Cognitive–behavioural change Self-management of pain	Problem-solving Reduce negative emotions Framing/reframing
Mindful attention control	To introduce Mindfulness as a tool to train attention and distract from pain	Principles of mind body therapies and biofeedback and visualisation	Behavioural practice Distraction Body changes
Balance and stretch	To promote body awareness and core strength	Guidelines on exercise Physical therapy principles	Demonstration of behaviour Behavioural practice
Summary of the day	To consolidate learning of the day and outline aims for final group day 3. A reminder to attend the one to one appointment with the clinical facilitator.	Acceptance and principles of self-efficacy	Action planning Verbal persuasion about capability

I-WOTCH group based Sessions day 3 (week 3)	Aims	Theoretical underpinnings	Behaviour change taxonomy
Reflections from day two	To understand and empathise with the group and ascertain current thoughts	Social learning Self-efficacy	Review of behaviour
Anger, irritability and frustration	Identifying reasons for negative emotions and implementing goal setting and action planning	Cognitive–behavioural change Theory of planned behaviour and reasoned action	Reduce negative emotions Goal setting Action planning
Relationships: getting the most from your healthcare team (part1)	To reflect on consulting behaviour and promote effective communication and constructive consultations	Biopsychosocial theory Theory of planned behaviour and reasoned action	Information about antecedents Instruction on how to perform a behaviour (communication skills)
Relationships (part 2) listening skills	To improve listening and communication skills	Biopsychosocial theory Theory of planned behaviour and reasoned action	Social support (emotional)
Managing setbacks and non-drug management techniques	To know what to do when experiencing a setback or a flare up	Cognitive–behavioural change Self-efficacy	Anticipated regret Focus on past success
Mindful distraction activity –colouring	To learn how to focus the mind away from pain thoughts and use of opioids	Principles of mind body therapies and biofeedback and visualisation	Behavioural practice Distraction Body changes
Stretch	To learn how to stretch muscles gently with low risk of injury and pain	Biopsychosocial theory Self-efficacy Principles of acceptance	Demonstration of behaviour Behavioural practice
Mindfulness of thoughts and senses	To learn how to apply mindfulness of thoughts by detaching emotion from reality, to appreciate ‘the now’	Principles of mind body therapies Biofeedback and visualisation	Distraction
Summary of the day	To consolidate the days learning.	Acceptance and principles of self-efficacy	Action planning
Summary of the course	To clarify learning from past three group days and motivation to continue with opioid reduction	Acceptance and principles of self-efficacy	Review of behaviour Verbal persuasion about capability

One to one session	Aim	Theoretical Underpinnings	Behaviour Change Taxonomy
Interaction one: face to face with clinical facilitator	To reflect on group learning days, agree tapering goals and generate tapering plan	Cognitive–behavioural change Motivational Interviewing	Goal setting behaviour Action planning Graded task Pros and cons
Interaction two: 30 min via telephone call with clinical facilitator	To reflect on progress and offer support during the tapering process	Cognitive–behavioural change Motivational Interviewing	Review behaviour Behavioural contract (adapted – as generated plan written) Social reward (congratulating on effort made and progress towards tapering-verbal)
Interaction three: 30 min via telephone with clinical facilitator	To reflect on progress and offer support during the tapering process	Cognitive–behavioural change Motivational Interviewing	Identification of self as role model (their own behaviour may be an example to others as they taper)
Interaction four: face to face with clinical facilitator	To reflect on progress so far and discuss goals for future	Cognitive–behavioural change Motivational Interviewing	Review behaviour Review outcome goal If applicable: discrepancy between current behaviour and goal feedback on behaviour Goal setting (behaviour) Goal setting (outcome) Action planning

### Feasibility testing

Funding from the Hambelton and Richmond Clinical Commissioning Group for a community pain management service allowed us to test the feasibility of the I-WOTCH intervention. Seven people were trained by the study team to deliver the intervention (three community team clinicians, two nurses and two volunteer patients). Two courses were observed by a member of the study team to evaluate how the course content was delivered and received by both the group facilitators and the group participants (five participants in total). Discussions included, what worked well, what did not work well, and whether participants felt that the aims and objectives of the programme were met and suggestions for changes.

The second stage of feasibility was part of the pilot phase of the randomised controlled trial and involved facilitator training for the trial. Two groups were delivered in Coventry. From both stages of feasibility testing, feedback was taken on board and adaptions implemented for the training (table 3) and course content and structure (table 4).

**Table 3:** Feedback and changes pilot phases I and II: training

Feedback (pilot phase I and II)—training and facilitator feedback	Changes implemented
Facilitators agreed it is useful to go through the manual step by step, to gain familiarity with each component and navigate through the different stages. They preferred this rather than going through generic topics.	We incorporated this information into the training and prior to a group being delivered, if needed the study team helped to arrange meetings between the facilitators.
Facilitators felt it would be useful for all material to be emailed prior to the training to allow time for familiarisation with the manual.	Throughout the I-WOTCH study all course materials were sent to facilitators prior to training.
Facilitators suggested that during the training it would be useful to actually practice some of the sessions.	Where possible during the training days we incorporated case studies and role play, as well as experiential learning of mindfulness and using the tapering app to calculate opioid reduction doses.
Facilitators suggested that it would be useful if the course slides were numbered in correspondence to the sections in the manual.	All course slides were numbered and added to the manual for reference.
Facilitators also suggested that it would be useful to include the rationale for each topic into the manual, as it helped with their understanding of each topic and with their explanation to participants.	The rationale for each topic was included in the manual.

I-WOTCH, Improving the Wellbeing of people with Opioid Treated CHronic pain.

**Table 4:** Feedback and changes pilot phases I and II: course content and structure

Feedback (pilot phase I and II) participant feedback	Changes implemented
During pilot phase I, feedback favoured spreading the group sessions over 3 weeks (one group day per week). This was to help with consolidation of information and learning between sessions and also felt less burdensome.	In the I-WOTCH study, groups were delivered with this format (every Monday where possible for 3 weeks).
It was suggested the balance session worked well after the session on posture, to allow more understanding and connection with body.	This was changed in the I-WOTCH programme: balance and stretch was introduced on day 2 of the programme and posture and movement on day 1 of the programme.
Day 1 presented a lot of educational information on opioids and it was suggested to split this over 2 days to help support consolidation of understanding	The educational information was split over 2 days (day 1 and day 2 of the programme).
It was also suggested to move the session on pacing to after the pain cycle has been discussed, to help with the understanding of why pacing is important and can help break the unhelpful cycle.	The pain cycle was introduced and on day 1 of the programme and pacing was moved to day two of the programme.
During pilot phase I, patients welcomed an educational DVD to help with the learning.	As part of the I-WOTCH study, we produced an I-WOTCH education DVD which is used in the delivery of the programme, participants are able to then take this home and watch with their family and friends or keep as a resource for themselves.

I-WOTCH, Improving the Wellbeing of people with Opioid Treated CHronic pain.

Overall, the feedback regarding the content of programme was positive. Participants felt that the distraction techniques worked well and helped break up the sessions. They also valued understanding the link between mood and pain and found the case studies useful in helping to motivate them to start reducing their opioids. Facilitators and participants in both pilot phases reported that it was an informative interactive course. Observations showed good delivery and interaction between facilitators and participants, good use of questions and answer sessions and role play. Both facilitators and patients agreed it may have been more interactive had the group been larger.

### Final I-WOTCH intervention

**Figure 3:**
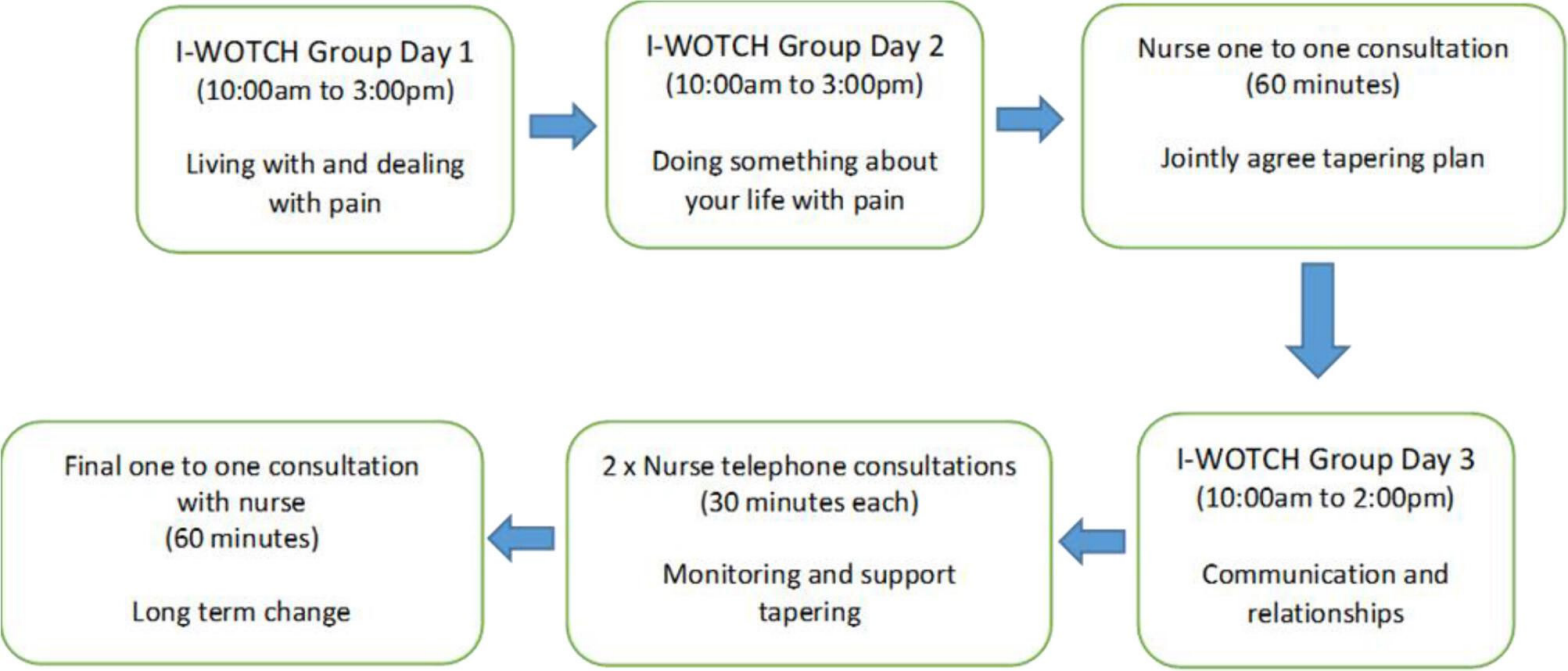
Final model of Improving the Wellbeing of people with Opioid Treated CHronic pain (I-WOTCH) intervention.

The final I-WOTCH intervention (figure 3) consists of group day 1 (delivered week 1), group day 2 (delivered week 2), a one-to-one consultation with an I-WOTCH trained nurse (also in week 2 and after group day 2), group day 3 (week 3) and then two telephone consultations and a final face-to-face consultation to offer continual support for tapering. Each component of the intervention builds on previous knowledge and experience, and where the one-to-one consultation allows consolidation and tailoring of advice and support for tapering. At the beginning of the intervention, the learning is centred on pain and opioid education, with day 2 of the programme then introducing changes in beliefs and adapting different strategies as reduction of opioids occur. It is at this point tailoring support and MI to action a change in beliefs is promoted through the one-to-one support sessions with an opportunity for further regulation and group cohesion/support on the wider impact of opioid reduction and long term behaviour change (group day 3). The additonal one to one support, self-regulation, reflection and monitoring.

### One-to-one consultations

The one-to-one sessions with a trained I-WOTCH nurse were based on a MI model.^27^ The aims of MI are to enhance behaviour change through a patient-centred framework, where the patient is able to explore personal goals, ambivalence to change and reach self-actualisation in a supportive environment. We trained the I-WOTCH nurses on the five principles of MI: (1) expressing empathy through reflective learning, (2) expressing empathy through reflective listening, (3) developing discrepancy between participant goals or values (related to opioid tapering and pain management) and their current behaviour, avoiding argument and direct confrontation, (4) adjusting to participant resistance to reducing opioid reduction rather than opposing it directly and (5) supporting self-efficacy and optimism. The one-to-one consultations included a review of medication, reflection on the opioid education and group session where case studies and information were presented and exploring any challenges to opioid tapering such as concerns about withdrawal. Nurses were also trained to calculate total opioid daily dose and how to use that to produce a tapering regime according to the I-WOTCH study protocol. Although MI has been widely applied in substance misuse, there are limited data available for its use in opioid cessation for people with chronic non-malignant pain. A 2020 pilot study testing MI to support opioid tapering in post joint arthroplasty surgery found a 62% increase in the rate of participants returning to baseline opioid use after surgery (HR 1.62; 95% CI 1.06 to 2.46; p=0.03).^28^ Opioid tapering conversations maybe challenging and each participant will bring their own experiences and motivation to change; however by using MI as a tool, we encouraged I-WOTCH facilitators to support participants in their tapering.^29^

### One-to-one tapering: App

We adopted an opioid tapering regimen based on the Mayo Clinic experience as it provided some evidence to support the notion that slow tapering is unlikely to be associated with severe withdrawal symptoms and therefore likely to facilitate adherence.^30^ This consisted of a 10% reduction of the original total daily dose every 7 days until a 30% of the original daily dose is reached. This is followed by a weekly decrease by 10% of the remaining dose. The 10% was rounded up to suit prescribing. For the calculation of equianalgesic doses, we used the tables from the Faculty of Pain Medicine.^31^ In order to ensure standardisation of tapering methodology across sites and various opioid preparations, the team developed a tapering App for use by the I-WOTCH trained nurses across sites. The I-WOTCH tapering App was developed by JN and SE working with the University of Warwick's Clinical Trials Unit (CTU) programming team (HA, CM and AW) and provided to the nurses on a handheld tablet. The I-WOTCH tapering App was based on a mathematical algorithm applying the Mayo Clinic tapering regime while accounting for UK commercial preparations. Nurses used the App to generate a participant specific tapering plan, which was synchronised to the I-WOTCH Trial database. The study team at Warwick CTU then logged into the centralised trial management website, printed and posted the tapering plan to the participant for their information and general practitioner for prescribing.

The I-WOTCH trained nurse entered the total daily dose of the participant-specific opioid preparation into the home screen of the App (eg, 60 mg oxycodone/day). The App algorithm then calculated 10% of the total daily dose and rounded this up or down to suit prescribing. All tablet, capsule or patch denominations of all opioid preparations were tabulated and added to the App to ensure the algorithm not only produced a 10% per week tapering regime but also recommended various prescribing methods (eg, oxycodone 35 mg could be prescribed as 30 and 5 mg or 20, 10 and 5 mg tablets or 10, 10, 10 and 5 mg tablets).

For patch preparations, we advised participants to taper using their original opioid if 10% was not achievable (eg, 12 μg of fentanyl being the smallest step down), the app algorithm was adjusted to recommend a 20% taper at 2-week intervals. Lowest dosage patch preparations were finally converted to slow release morphine equianalgesic doses and tapered accordingly.

### My opioid manager

The My Opioid Manager Book and App is the output of a project of Toronto Rehabilitation Institute, University Health Network. In 2010, Dr. Andrea Furlan, a Physician and Scientist at Toronto Rehabilitation Institute, developed a tool for physicians prescribing opioids for patients with chronic non-malignant pain. In 2012, the Opioid Manager was converted to an App for smartphones and tablets. The My Opioid Manager Book (and App) is intended to complement the Opioid Manager by providing the same information in a format that can be used by people with chronic pain who are on opioids, or by people who are not on opioids but who might be considering this option to help manage their chronic pain. The goal of My Opioid Manager is preparing the patient for upcoming consultations with their healthcare provider. Some of the topics discussed include: understanding the causes of various types of pains, uses of opioids and the side effects and risks, managing pain by tracking opioid usefilness, and tips on using opioids. For this study, we Anglicised the content in terms of language used as well as the name of medication brands and pictures to be more representative of the UK population.

### Venue for delivering the intervention

Where possible, the I-WOTCH intervention is delivered in the community. Factors to consider when booking a venue included, access to a building, parking and public transport links, a room to accommodate participants and facilitators with chairs and equipment, stairs, lifts, kitchen facilities and room for equipment such as a flipchart, laptop screen, speakers and internet access.

### I-WOTCH facilitator training

The delivery–receipt–enactment chain of the I-WOTCH intervention provided a framework for training of facilitators and defining dosage received for participants to promote behaviour change (opioid tapering).^32^ The I-WOTCH training included two full days for all facilitators (clinical and lay facilitators) and an additional day for clinical facilitators only, to learn the clinical aspects of tapering, opioid specific education, generating tapering plans and MI for the one-to-one consultations. The design of the training package and implementation was adapted to Kolb’s experiential learning cycle (training, experience and reflective observation).^33^ The training days gave all facilitators exposure to the different components of the intervention through education and use of case studies. Trainers were given copies of the I-WOTCH manual and all participant intervention materials. Throughout the training days, facilitators had the opportunity to ask questions and get clarity on any of the topics being covered. At the end of the training, a short assessment was completed by each trainee. If any of the trainees scored below 70%, they were then contacted by phone to go over any areas needing further explanation and offered further training if needed.

## Discussion

We have used a theory driven approach to developing an intervention for opioid reduction for people with chronic non-malignant pain. Based on the COPERS intervention for the management of pain, the best available empirical evidence at the time, and consultation with lay people, we have developed a manualised intervention and training package. It has been piloted, revised and adapted considering all feedback received. The I-WOTCH intervention has the potential to help people reduce their opioid use and improve their overall quality of life. We are not aware of any other programme of analogous interventions targeting similar populations. Previous non-pharmacological interventions have included mindfulness, cognitive–behavioural therapy and meditation and the use of electroacupuncture which showed no reduction in the number of participants who ceased their opioid use.^4^ The I-WOTCH intervention differs in that it combines group and one to one support, with the mechanisms of change and opioid reduction targeted through peer support, education, case studies, reflection and MI. It is a time and resource intensive intervention, however, having a multicomponent intervention will increase the potential to address the complex psychological, social and physical aspects of opioid tapering. We have developed an opioid tapering App which can be used to calculate individual opioid tapering plans.

The roll out and scalability of the I-WOTCH training have been considered, a step-by-step manual with materials to set up and deliver the programme was created. The I-WOTCH facilitator training can be delivered to groups of clinicians and ongoing support given throughout the delivery of the intervention. The I-WOTCH trial will allow us to assess: the delivery of the intervention on a large scale, the training of multiple facilitators and managing the group element of the programme.

## Conclusion

We have designed an opioid reduction intervention package suitable for testing in a randomised controlled trial.

## Supplementary Material

Reviewer comments

Author's manuscript

## Data Availability

No data are available. No additional data available.
