# Supplementary material for: Development and testing of an opioid tapering self-management intervention for chronic pain: I-WOTCH
Source: BMJ Open. 2022 Mar 16;12(3):e053725. doi: 10.1136/bmjopen-2021-053725 (PMC8928279; doi:10.1136/bmjopen-2021-053725)
Supplement: Author's manuscript [file bmjopen-2021-053725.draft_revisions.pdf]

BMJ Open

BMJ Open is committed to open peer review. As part of this commitment we make the peer review history of every article we publish publicly available.

When an article is published we post the peer reviewers' comments and the authors' responses online. We also post the versions of the paper that were used during peer review. These are the versions that the peer review comments apply to.

The versions of the paper that follow are the versions that were submitted during the peer review process. They are not the versions of record or the final published versions. They should not be cited or distributed as the published version of this manuscript.

BMJ Open is an open access journal and the full, final, typeset and author-corrected version of record of the manuscript is available on our site with no access controls, subscription charges or pay-per-view fees (http://bmjopen.bmj.com).

If you have any questions on BMJ Open's open peer review process please email info.bmjopen@bmj.com

BMJ Open

The development and testing of an opioid tapering self-management intervention for long term pain – I-WOTCH

Journal:	*BMJ Open*
Manuscript ID	bmjopen-2021-053725
Article Type:	Original research
Date Submitted by the Author:	21-May-2021

Complete List of Authors:	Sandhu, Harbinder; University of Warwick Warwick Medical School, Clinical Trials Unit<br>Shaw, Jane; James Cook University Hospital<br>Carnes, Dawn; Queen Mary University of London Barts and The London School of Medicine and Dentistry, Institute of Population Health Sciences<br>Furlan, Andrea; Toronto Rehabilitation Institute; University of Toronto Faculty of Medicine<br>Tysall, Colin; University of Warwick, UNTRAP; Coventry University, SUCE<br>Adjei, Henry; University of Warwick Warwick Medical School, Warwick Clinical Trials Unit<br>Muthiah, Chockalingam; University of Warwick, Warwick Clinical Trials Unit<br>Noyes, Jennifer; James Cook University Hospital, Department of Pain Medicine<br>Tang, Nicole; University of Warwick, Psychology<br>Taylor, Stephanie; Queen Mary University of London Barts and The London School of Medicine and Dentistry, Institute of Population Health Sciences<br>Underwood, Martin; University of Warwick Warwick Medical School, Warwick Clinical Trials Unit<br>Willis, Adrian; University of Warwick, Warwick Clinical Trials Unit<br>Eldabe, Sam; James Cook University Hospital, Department of Pain Medicine

Keywords:	Pain management < ANAESTHETICS, Rehabilitation medicine < INTERNAL MEDICINE, MEDICAL EDUCATION & TRAINING, PAIN MANAGEMENT, PRIMARY CARE, Clinical trials < THERAPEUTICS

SCHOLARONE™
Manuscripts

I, the Submitting Author has the right to grant and does grant on behalf of all authors of the Work (as defined in the below author licence), an exclusive licence and/or a non-exclusive licence for contributions from authors who are: i) UK Crown employees; ii) where BMJ has agreed a CC-BY licence shall apply, and/or iii) in accordance with the terms applicable for US Federal Government officers or employees acting as part of their official duties; on a worldwide, perpetual, irrevocable, royalty-free basis to BMJ Publishing Group Ltd ("BMJ") its licensees and where the relevant Journal is co-owned by BMJ to the co-owners of the Journal, to publish the Work in this journal and any other BMJ products and to exploit all rights, as set out in our licence.

The Submitting Author accepts and understands that any supply made under these terms is made by BMJ to the Submitting Author unless you are acting as an employee on behalf of your employer or a postgraduate student of an affiliated institution which is paying any applicable article publishing charge ("APC") for Open Access articles. Where the Submitting Author wishes to make the Work available on an Open Access basis (and intends to pay the relevant APC), the terms of reuse of such Open Access shall be governed by a Creative Commons licence – details of these licences and which Creative Commons licence will apply to this Work are set out in our licence referred to above.

Other than as permitted in any relevant BMJ Author's Self Archiving Policies, I confirm this Work has not been accepted for publication elsewhere, is not being considered for publication elsewhere and does not duplicate material already published. I confirm all authors consent to publication of this Work and authorise the granting of this licence.

For peer review only

The development and testing of an opioid tapering self-management intervention for long term pain – I-WOTCH

Harbinder Kaur Sandhu, Jane Shaw, Dawn Carnes, Andrea Dompieri Furlan, Colin Bernard Tysall, Henry Adjei, Chockalingam Muthiah, Jennifer Noyes, Nicole K Y Tang , Stephanie JC Taylor, Martin Underwood, Adrian Willis, Sam Eldabe, On behalf of the I-WOTCH Team

Harbinder Kaur Sandhu, Warwick Clinical Trials Unit, Warwick Medical School, University of Warwick, Coventry CV4 7AL, UK

Harbinder.K.Sandhu@warwick.ac.uk

Jane Shaw, The James Cook University Hospital, Middlesbrough, TS4 3BW, UK

Jane@janeshaw.net

Dawn Carnes PhD, Institute of Population Health Sciences, Barts and The London School of Medicine and Dentistry, Queen Mary University of London, UK

d.carnes@qmul.ac.uk

Andrea Dompieri Furlan, Toronto Rehabilitation Institute, University Health Network, Department of Medicine, University of Toronto, Institute for Work & Health, Toronto Canada

Andrea.furlan@uhn.ca

Colin Bernard Tysall, UNTRAP Warwick University, SUCE Coventry University, UK

colin@tysall.co.uk

For peer review only - http://bmjopen.bmj.com/site/about/guidelines.xhtml

Henry Adjei, Warwick Clinical Trials Unit, Warwick Medical School, University of Warwick, Coventry CV4 7AL, UK

H.Adjei@warwick.ac.uk

Chockalingam Muthiah, Warwick Clinical Trials Unit, Warwick Medical School, University of Warwick, Coventry CV4 7AL, UK

c.muthiah@warwick.ac.uk

Jennifer Noyes, Department of Pain Medicine, Murray Building, The James Cook University Hospital, Middlesbrough, TS4 3BW, UK

j.noyes@nhs.net

Nicole K Y Tang, Department of Psychology, University of Warwick, Coventry CV4 7AL, UK

N.Tang@warwick.ac.uk

Stephanie JC Taylor, Institute of Population Health Sciences, Barts and The London School of Medicine and Dentistry, Queen Mary University of London, UK

s.j.c.taylor@qmul.ac.uk

Martin Underwood, Warwick Clinical Trials Unit, Warwick Medical School, University of Warwick, Coventry CV4 7AL, UK and University Hospitals Coventry and Warwickshire, Clifford Bridge Road Coventry CV2 2DX, UK

M.Underwood@warwick.ac.uk

For peer review only

Adrian Willis, Warwick Clinical Trials Unit, Warwick Medical School, University of Warwick, Coventry CV4 7AL, UK

Adrian.Willis@warwick.ac.uk

Sam Eldabe, Department of Pain Medicine, Murray Building, The James Cook University Hospital, Middlesbrough, TS4 3BW, UK

seldabe@nhs.ne

Corresponding Author: Harbinder Kaur Sandhu, Warwick Clinical Trials Unit, Warwick Medical School, University of Warwick, Coventry CV4 7AL, UK

Harbinder.K.Sandhu@warwick.ac.uk

Abstract

Aims and objectives: The I-WOTCH intervention is designed to support people with chronic non-malignant pain to withdraw from opioids using education, group cohesion, problem solving, motivation, one to one tailored planning and monitoring and reflection to enhance and encourage self-management of pain.

Methods: The theoretical basis of the I-WOTCH intervention included the design of complex interventions (The Medical Research Council Framework) behaviour change framework and psychological theories to support mechanisms of behaviour change, linking components of the intervention together and overall content and structure of the programme. The I-WOTCH intervention was based on previous work of self-management of chronic pain (COPERS).

For peer review only - http://bmjopen.bmj.com/site/about/guidelines.xhtml

Results: Based on previous literature on self-management of pain, opioid withdrawal and feedback from two PPI meetings (n=19) as part of the North East and North Cumbria Clinical Research Network, we were able to adapt and develop content and structure of the I-WOTCH Intervention. Feedback included the target behaviour change to be reduction in opioid consumption and engagement with the I-WOTCH programme. Motivation was also agreed to be important and the use of case studies to demonstrate successful opioid withdrawal and education on reduction of side effects and having a "'trade-off' encouraging other strategies to manage pain (in this case self-management). The intervention is delivered by a trained I-WOTCH clinician and a lay facilitator with experience of opioid reduction. After piloting the final I-WOTCH structure was agreed including a detailed facilitator manual to deliver the intervention; participant material including handout, an educational DVD and relaxation and mindfulness CD; My Opioid Manager (adapted); and a tapering App for clinical facilitators that generated a tailored tapering plan for each participant.

Conclusions: We have designed an opioid reduction intervention package suitable for testing in a randomised controlled trial.

Article Summary: Strengths and limitations

1. The I-WOTCH intervention is based on theoretical underpinning.

2. The I-WOTCH intervention content and structure was designed with input from patient and public involvement.

3. A training package to deliver the I-WOTCH Intervention for facilitators was developed and piloted.

4. At the time of designing the intervention there was limited previous work and information to inform content of the intervention.

For peer review only - http://bmjopen.bmj.com/site/about/guidelines.xhtml

Key words: Opioid withdrawal, medication reduction, behaviour change, chronic non cancer pain, self-management, intervention development

Total word count: 3,060

Introduction

Pain, and pain related disorders, continue to be the leading cause of disability and disease burden globally (1), with low back pain reported as the leading cause of years lived with disability. In England at least eight million people (15% of the population) have moderate to severe persistent (chronic) pain (2) defined as *pain that lasts or recurs for more than three months.*(3) Over the past few decades, there has been a global epidemic of opioid prescribing for chronic non cancer pain. A 2020 systematic review found that 30 percent of people with chronic non-malignant pain are prescribed opioid medication and, globally, this has steadily increased until recently with time. (4) In the UK prescribing rates have decreased slightly over recent years, however the number of prescriptions still remains high.(5) Long term use of opioids can lead to tolerance and result in the loss of effective pain relief. Adverse consequences include opioid induced hyperalgesia, endocrine disorders and hypogandism, drowsiness, a high risk of dependency, opioid use disorder, sleep apnoea, immune suppression, and falls leading to increased fractures (particularly a risk in the elderly population), and death.(6) There is also an increased risk for overdose and potential for sexual dysfunction, with limited strategies to help with risk mitigation and interventions to help people with chronic non-malignant pain withdraw from opioids.(7) A 2020 systematic review evaluating the efficacy of opioid de-prescribing interventions in randomised controlled trials for patients with chronic non-cancer pain found ten patient focused RCT interventions and two clinician focused interventions. However, the authors were unable to recommend any particular deprescribing strategy due to the small nember of studies and heterogeneous data.(4)

For peer review only - http://bmjopen.bmj.com/site/about/guidelines.xhtml

Current recommendations on opioid tapering are based on best practice and guidelines which need to be supported by further evidence.(8) The current paper describes the development of a multi-component opioid tapering programme (incorporating group and one to one sessions) as part of the I-WOTCH study (Improving the Wellbeing of Opioid Treated Chronic Pain), funded by the National Institute of Health Research [14/224/04] This paper complements the study protocol paper.(9)

Methods

The I-WOTCH intervention was developed in collaboration with the target population (those with chronic non-malignant pain and experience of opioid use), and employed theory and evidence based implementation (with the view of implementation in the real world should it be effective) and included digital technologies to generate opioid tapering plans.(10) The Medical Research Council Framework (11) for designing complex interventions and core theoretical principles was used to inform content, structure, and delivery of the intervention.

Key stages of the intervention development are outlined in figure 1. Adjustment and adaptation to the intervention were implemented in line with feedback received from stakeholders (service users, clinicians and facilitators delivering the I-WOTCH intervention).

Aims and objectives of the I-WOTCH intervention

In line with the overall study, the aims of the I-WOTCH intervention were to:

1) To reduce opioid and healthcare use for people with chronic non-malignant pain
2) To increase self-efficacy (confidence to reduce opioid medication and implement self-management strategies of pain)
3) To improve quality of life and help people live better with pain

For peer review only - http://bmjopen.bmj.com/site/about/guidelines.xhtml

Objectives:

1) To provide education using a range of teaching methods; group discussion, problem solving, experiential learning and case studies.
2) To provide an environment which enhances motivation through group cohesion and one to one support.
3) To provide an overall cost-effective intervention to be implemented in healthcare services.

Defining the aims and objectives enabled us to consider what we wanted to achieve, how and for what purpose. In addition, we were aware of potential facilitators and barriers that could influence engagement with the intervention and the procedures of the trial. Figure 2 shows the direction of travel we were aiming for and what we needed to consider when designing the detail of intervention and mechanism of behaviour change.

Patient and public involvement

During the development stages of I-WOTCH, we held two PPI meetings with the Clinical Research Network (North East and Cumbria) at The James Cook University Hospital (South Tees Hospitals NHS Foundation Trust). A total of nineteen volunteer participants (people with chronic pain and experience of opioid therapy and/or opioid tapering) attended. Discussions were facilitated by members of the study team (HS, DC, JS and SE) and included, intervention structure and design, content (topics to cover which would potentially increase motivation and confidence to taper opioids), length of programme, where the intervention should be delivered, support during opioid tapering (including frequency of contact with healthcare professionals) and delivery of the intervention (who should deliver the intervention) (Table 1). In addition to this, two lay advisors who were apart of the I-WOTCH study recruited via Universities/User Teaching, (13) gave considerable input into the design and training of the intervention.

For peer review only - http://bmjopen.bmj.com/site/about/guidelines.xhtml

Table 1: Feedback from PPI Informing Intervention Development

Discussion topic	Feedback informing intervention development
Behaviour change	Agreed aims should be a reduction in opioid consumption and engagement in the I-WOTCH programme.<br><br>Behaviour change needs to be accepted before opioid reduction can occur.
Understanding motivation to change behaviour	Changing medication and reducing medication can be motivated by:<br><br>i) a trade-off to fill the deficit of the effect of the drug (something else needed that is as effective as the drug they would lose)<br>ii) reduction in side effects<br><br>Use of case studies of people who had stopped taking opioids and that were successful would be useful.
Content and topics to be covered	The intervention would benefit from being informative (opioid education, especially long term consequences, pros and cons of opioid use and managing withdrawal).<br><br>The following topics were recommended to be included:<br>• What is Pain<br>• Acceptance – pain and learning to live better with pain<br>• Impact of pain – and integrate this information with taking medication (Opioids), why and how?<br>• The importance of hobbies and having a distraction to manage the pain<br>• Offer alternative non-pharmacological ways of coping, e.g. mindfulness and relaxation<br>• Incorporate movement<br>• Guidance on posture and exercise/activity<br>• Pacing – not over doing things
Dependency vs addiction	It was felt important to distinguish between dependency and addiction, as some were concerned about the stigma and label attached to opioid users for long term pain.
Delivery of I-WOTCH Intervention, who?	Feedback favoured the course to be delivered by a HCP and lay facilitator someone who had experience of long term pain and opioid use/tapering.
Structure of Intervention	Group and individual care approaches were valued.<br>Length of the proposed programme (3-day group sessions and ongoing one to one support) was supported.<br>The duration of intervention was not viewed as burdensome given that some had people who have experienced severe withdrawal symptoms, and therefore ongoing support over the 8 to 10 weeks is needed.

For peer review only - http://bmjopen.bmj.com/site/about/guidelines.xhtml

	There was a consensus that a group-based format would be optimal because of the potential for social comparison, social validation and development of social support within a group setting. Volunteers identified the impact of opioid use on enhanced day-to-day activities as important evaluation outcomes, including: work productivity, looking after children, and overall functioning.
Communication during study	Volunteers welcomed the idea of having a study website to give participants an opportunity to be updated about the study as a whole and progress.

Opioid Tapering and Behaviour Change

The target behaviour change was defined as the participants engaging in the I-WOTCH intervention, reducing participant opioid use, and implementing non-pharmacological strategies of pain management. The bio-psychosocial framework (14) and Michie's taxonomy of behaviour change was consulted, in particular the COM-B model (Capability, Opportunity, Motivation).(15) Capability includes psychological capability (can patients engage in the necessary thought processes needed to engage in the tapering processes?) and physical capability (do participants have the capacity to engage in the tapering?). Psychological capability is broken down to cognitive functioning and executive functioning. To promote cognitive functioning (which includes a range of mental abilities such as learning, problem solving and attention) we produced handouts of material covered on each day the programme. This allowed opportunity for participants to recap over the core messages and information in their own time. We also included time for group reflection at the start of each session and summarising discussions at the end of each of the group days (with opportunities for questions). In addition to this we developed an educational DVD, a mindfulness CD and relaxation CD for each participant. By providing material to take home we were giving participants an opportunity to revisit and take in the information at their own pace.(16) Executive functioning includes the capacity to plan and think, explore challenges that may occur (for example fear of withdrawal symptoms), stay focused on the goal (opioid reduction) and resist temptation.(17)

For peer review only - http://bmjopen.bmj.com/site/about/guidelines.xhtml

In the I-WOTCH intervention we gave participants opportunity to set goals (through an educational session and support in generating goals related to opioid tapering and their general life). We also encouraged self-reflection to identify perceived barriers and facilitators to tapering and gave further guidance to overcome the perceived barriers in the tailored one to one support sessions with the clinical facilitator. Physical capability refers to whether the participants exposed to the I-WOTCH Intervention felt they had the right skills to engage in the tapering of their opioids, this may include management of withdrawal, confidence and having structure and support in place. The I-WOTCH intervention was designed to help participants adapt and put into place lifestyle changes.

Opportunity is the second component of the COM-B model. For this we explored factors external to the individual that would promote opioid tapering. For example, physical opportunity which includes, costs of opioids and travel, access and availability, developing a tapering plan (being clear and informative of who and how this would be developed) and enhancing communication between the clinical facilitator and participant through motivational interviewing during the tapering processes. Social opportunity, we referred to what other factors may impact the decision to taper such as stigma and cultural beliefs.

Motivation, this refers to both the cognitive motivation and emotional processes to energise and direct the behaviour change of opioid tapering. Reflective processes included exploring perceptions and meaning of chronic pain during the group sessions as well as beliefs about tapering, possible outcomes concerns and self-efficacy. There was opportunity to evaluate and be reflective during the group sessions as well as one to one support. Automatic processes refer to the emotional responses which may occur during the I-WOTCH intervention and these include anxiety, fear, stress, and low mood. All topics were covered in the group sessions including recognition of thoughts and emotions and management strategies.

For peer review only - http://bmjopen.bmj.com/site/about/guidelines.xhtml

Each component of the I-WOTCH intervention was informed and mapped on to behaviour change taxonomy's (BCTv1). The intervention also drew on psychological theories of self-efficacy,(18) Theory of planned behaviour and reasoned action,(19, 20) social learning (21) and group based interventions,(22) cognitive behaviour-change, (23) motivational interviewing (24) and evidence based interventions for self-managing chronic pain (COPERS) (25) described in Table 2.

For peer review only - http://bmjopen.bmj.com/site/about/guidelines.xhtml

Table 2: Behaviour change taxonomy and opioid tapering

I-WOTCH Group based sessions day 1 (week 1)	Aims	Theoretical Underpinnings	Behaviour Change Taxonomy
Introductions, group work, aims	To allow participants to introduce themselves to the group, encourage participation in a safe and relaxed environment, explore expectations and discuss the I-WOTCH course aims	Social cognitive theory, Bio-psychosocial theory	Improve bonding and group cohesion. Breaking barriers and encouraging self and social awareness
What Causes Pain? (Pain information)	To increase understanding about long-term pain	Biopsychosocial theory<br>Principles of self-efficacy and acceptance	Credible Source
Living With Pain (Opioid education I)	To increase understanding about use of opioids for long term pain and encourage participants to start questioning their own knowledge and beliefs about opioids and why they take them	Biopsychosocial theory<br>Theory of planned behaviour and reasoned action<br>Health Beliefs	Information about health consequences
Acceptance	To understand and start to accept pain, with a view to implementing self-management strategies as reduction of opioids occurs	Acceptance and Self-management of chronic pain	Goal setting<br>Commitment
Attention Control & Distraction	To learn how to focus the mind away from pain thoughts and use of opioids	Cognitive behaviour change<br>Self-management of chronic pain<br>Health beliefs	Distraction
Distraction activity – drawing	An opportunity to practice distraction activity and socially interact with group informally	Cognitive behaviour change<br>Social learning	Behavioural practice<br>Distraction
Good days, bad days - Pain, bearable or not?	To reinforce that pain is not just	Biopsychosocial theory	Information and antecedents

For peer review only - http://bmjopen.bmj.com/site/about/guidelines.xhtml

	physiological, it is psychological, social and an emotional phenomenon	Health beliefs	Information about health consequences<br>Re-attribution of behaviour
The pain cycle (including opioids) and breaking the pain cycle	To explain and identify unhelpful factors in the pain cycle and learn strategies to break the cycle	Biopsychosocial Theory<br>Health beliefs	Behaviour Substitution (adding in other behaviours to break cycle)
Posture and movement	To promote body awareness, posture and muscle weakness (Managing pain without opioids)	Theory of planned behaviour and reasoned action	Guidelines on exercise, physical therapy principles, Mindfulness
Relaxation and breathing	To reduce muscle tension and introduce breathing as a relaxation technique	Cognitive behaviour change<br>Self-management of chronic pain	Behavioural practice<br>Distraction<br>Body changes
Summary of the day	To consolidate learning of the day and outline aims for group day 2.	Acceptance and principles of self-efficacy	Action planning<br>Verbal persuasion about capability
I-WOTCH group based Sessions Day 2 (week 2)	Aims	Theoretical Underpinnings	Behaviour Change Taxonomy
Reflections from day 1	To understand and empathise with the group	Social learning<br>Self-efficacy	Improve bonding and group cohesion, social cognitive theory
Stress-busting for Health: Action planning, problem solving, pacing, SMART goal setting	To help the participants logically and systematically identify problems, free think solutions, set achievable goals and create action plans, as a means of escaping the pain cycle	Cognitive behaviour change<br>Theory of planned behaviour and reasoned action	Goal setting<br>Comparative imagining of future outcomes<br>Reduce negative emotions<br>Problem solving
Withdrawal symptoms, Case studies (Opioid education II)	To discuss potential withdrawal symptoms that participants might experience if their taper is too quick	Health beliefs<br>Social learning	Social comparison (drawing attention to others' performance to allow comparison with the person's own performance)<br>Credible source<br>Comparative imagining of future outcomes

For peer review only - http://bmjopen.bmj.com/site/about/guidelines.xhtml

Distraction activity – origami	To learn how to focus the mind away from pain thoughts and use of opioids	Cognitive behaviour change<br>Social learning	Behavioural practice<br>Distraction
Identifying and overcoming barriers to change	Introduce ideas about unhelpful thoughts, automatic thoughts and errors in thinking. To identify reasons why people stay in the pain cycle, and barriers to change. Introduce positive reframing	Cognitive behaviour change<br>Self-management of pain	Problem solving<br>Reduce negative emotions<br>Framing/reframing
Mindful attention control	To introduce Mindfulness as a tool to train attention and distract from pain	Principles of mind body therapies and biofeedback and visualisation	Behavioural practice<br>Distraction<br>Body changes
Balance and stretch	To promote body awareness and core strength	Guidelines on exercise, physical therapy principles	Demonstration of behaviour<br>Behavioural practice
Summary of the day	To consolidate learning of the day and outline aims for final group day 3. A reminder to attend the one to one appointment with the clinical facilitator.	Acceptance and principles of self-efficacy	Action planning<br>Verbal persuasion about capability
I-WOTCH group based Sessions Day 3 (week 3)	Aims	Theoretical Underpinnings	Behaviour Change Taxonomy
Reflections from day two	To understand and empathise with the group and ascertain current thoughts	Social learning<br>Self-efficacy	Review of behaviour
Anger, irritability and frustration	Identifying reasons for negative emotions and implementing goal setting and action planning	Cognitive behaviour change<br>Theory of planned behaviour and reasoned action	Reduce negative emotions<br>Goal setting<br>Action planning
Relationships: Getting the most from your healthcare team (Part1)	To reflect on consulting behaviour and promote effective communication and constructive consultations	Biopsychosocial theory<br>Theory of planned behaviour and reasoned action	Information about antecedents<br>Instruction on how to perform a behaviour (communication skills)
Relationships (Part 2) Listening skills	To improve listening and communication skills	Biopsychosocial theory<br>Theory of planned behaviour and	Social support (emotional)

For peer review only - http://bmjopen.bmj.com/site/about/guidelines.xhtml

		reasoned action	
Managing setbacks and non-drug management techniques	To know what to do when experiencing a setback or a flare up	Cognitive behaviour change<br>Self-efficacy	Anticipated regret<br>Focus on past success
Mindful distraction activity –colouring	To learn how to focus the mind away from pain thoughts and use of opioids	Principles of mind body therapies and biofeedback and visualisation	Behavioural practice<br>Distraction<br>Body changes
Stretch	To learn how to stretch muscles gently with low risk of injury and pain	Biopsychosocial theory<br>Self-efficacy<br>Principles of acceptance	Demonstration of behaviour<br>Behavioural practice
Mindfulness of Thoughts & Senses	To learn how to apply mindfulness of thoughts by detaching emotion from reality, to appreciate 'the now'	Principles of mind body therapies and biofeedback and visualisation	Distraction
Summary of the day	To consolidate the days learning.	Acceptance and principles of self-efficacy	Action planning
Summary of the course	To clarify learning from past 3 group days and motivation to continue with opioid reduction	Acceptance and principles of self-efficacy	Review of behaviour<br>Verbal persuasion about capability
**One to one session**	**Aim**	**Theoretical Underpinnings**	**Behaviour Change Taxonomy**
Interaction one: face to face with clinical facilitator	To reflect on group learning days, agree tapering goals and generate tapering plan	Cognitive behaviour change<br>Motivational Interviewing	Goal setting behaviour<br>Action planning<br>Graded task<br>Pros and cons
Interaction two: 30 minute via telephone call with clinical facilitator	To reflect on progress and offer support during the tapering process	Cognitive behaviour change<br>Motivational interviewing	Review behaviour<br>Behavioural contract (adapted – as generated plan written)<br>Social reward (congratulating on effort made and progress towards tapering-verbal)
Interaction three: 30 minute via telephone with	To reflect on progress and offer support during the	Cognitive behaviour change<br>Motivational	Identification of self as role model (their own behaviour may

For peer review only - http://bmjopen.bmj.com/site/about/guidelines.xhtml

clinical facilitator	tapering process	interviewing	be an example to others as they taper)
Interaction four: face to face with clinical facilitator	To reflect on progress so far and goals and discuss goals for future	Cognitive behaviour change<br>Motivational interviewing	Review behaviour<br>Review outcome goal<br>If applicable: discrepancy between current behaviour and goal<br>Feedback on behaviour<br>Goal setting (behaviour)<br>Goal setting (outcome)<br>Action planning

Feasibility Testing

Funding from the Hambelton and Richmond Clinical Commissioning Group for a community pain management service allowed us to test the feasibility of the I-WOTCH Intervention. Seven people were trained by the study team to deliver the intervention (3 community team clinicians 2 nurses and 2 volunteer patients). Two courses were observed to evaluate how the course content was delivered and received by both the group facilitators and the group participants (five participants in total). Discussions included, what worked well, what did not work well, and whether participants felt that the aims and objectives of the programme were met and suggestions for changes.

The second stage of feasibility was as part of the pilot phase of the randomised controlled trial and involved facilitator training for the trial. Two groups were delivered in Coventry. From both stages of feasibility testing feedback was taken on board and adaptions implemented for the training (Table 3) and course content and structure (Table 4)

Table 3: Feedback and changes pilot phases I and II- Training

For peer review only

Feedback (Pilot phase I and II) – Training and facilitator feedback	Changes implemented
Facilitators agreed it is useful to go through the manual step by step, to gain familiarity with each component and navigate through the different stages. They preferred this rather than go through generic topics.	We incorporated this information into the training and prior to a group being delivered if needed the study team helped to arrange meetings between the facilitators.
Facilitators felt it would be useful to all material emailed prior to the training to allow time to read and become familiar with the manual.	Throughout the I-WOTCH study all course material was sent to facilitators prior to training.
Facilitators suggested that during the training it would be useful to actually practice some of the sessions.	Where possible during the training days we incorporated case studies, role play as well as experiential learning of mindfulness and using the tapering app to calculate opioid reduction doses.
Facilitators suggested that it would be useful if the course slides were numbered in correspondence to the sections in the manual.	All course slides numbered and added to manual as reference.
Facilitators also suggested that it would be useful to include rational of each topic into the manual, as it helps with their understanding of each topic and explanation to participants.	Rational for each topic was included in the manual.

Table 4: Feedback and changes pilot phases I and II- Course content and structure

Feedback (Pilot phase I and II) participant feedback	Changes implemented
During pilot phase I, feedback favoured to spread the group sessions over three weeks (one group day a week). This was to help with consolidation of information and learning between sessions and also felt less burdensome.	The I-WOTCH group structure then through the study was delivered within the format of one group session a week (every Monday for three weeks).
It was suggested the balance session works well after posture, to allow more understanding and connection with body.	This was changed in the I-WOTCH programme, balance and stretch was introduced on day 2 of the programme and posture and movement on day 1 of the programme.
Day 1 presented with a lot of opioid educational information and it was suggested to help with understanding of this topic to split them over two days.	The opioid educational information was split over two days (day 1 and day 2 of the programme).
It was also suggested to move pacing after the pain cycle has been discussed, to help with the understanding of why pacing is important and can help break the unhelpful cycle.	The pain cycle was introduced and on day 1 of the programme and pacing was moved to day two of the programme.

For peer review only - http://bmjopen.bmj.com/site/about/guidelines.xhtml

During Pilot phase I, patients welcomed an educational DVD to help with the learning.	As part of I-WOTCH we produced an I-WOTCH education DVD which is used in the delivery of the programme, participants are able to then take this home and watch with their family and friends or keep as a resource for themselves.

Overall, the feedback regarding the content of programme was positive. Participants felt that the distraction techniques worked well and helped break up the sessions. They also valued understanding the link between mood and pain and found the case studies useful in helping to motivate them to start their own journey of reducing their opioids. Facilitators and participants in both pilot phases reported that it was an informative interactive course. Observations showed good delivery and interaction between facilitators and participants, good use of questions and answer session and role play. Both facilitators and patients agreed it may have been more interactive with a larger group

Final I-WOTCH Intervention

The final I-WOTCH Intervention was agreed based on feedback and piloting. (Fig 2) it consists of group day 1 (delivered week one), group day two (delivered week two), a one to one consultation with the an I-WOTCH trained nurse (also in week 2 and after group day two), group day three (week three) and then two telephone consultations and final and a final face to face consultation to offer continual support for tapering. Each component of the intervention builds on previous knowledge and experience, and where the one to one consultation allows consolidation and tailoring of advice and support for tapering. At the beginning of the intervention the learning is centred on pain and opioid education, with day two of the programme then introducing changes in beliefs and adapting different strategies as reduction of opioids occur. It is at this point tailoring support and motivational interviewing to action a change in beliefs is promoted through the one to one support sessions with an opportunity for

For peer review only - http://bmjopen.bmj.com/site/about/guidelines.xhtml

further regulation and group cohesion/support on the wider impact of opioid reduction and long term behaviour change. The further one to one support promotions, self-regulation, reflection and monitoring.

Figure 2: Final model of I-WOTCH Intervention

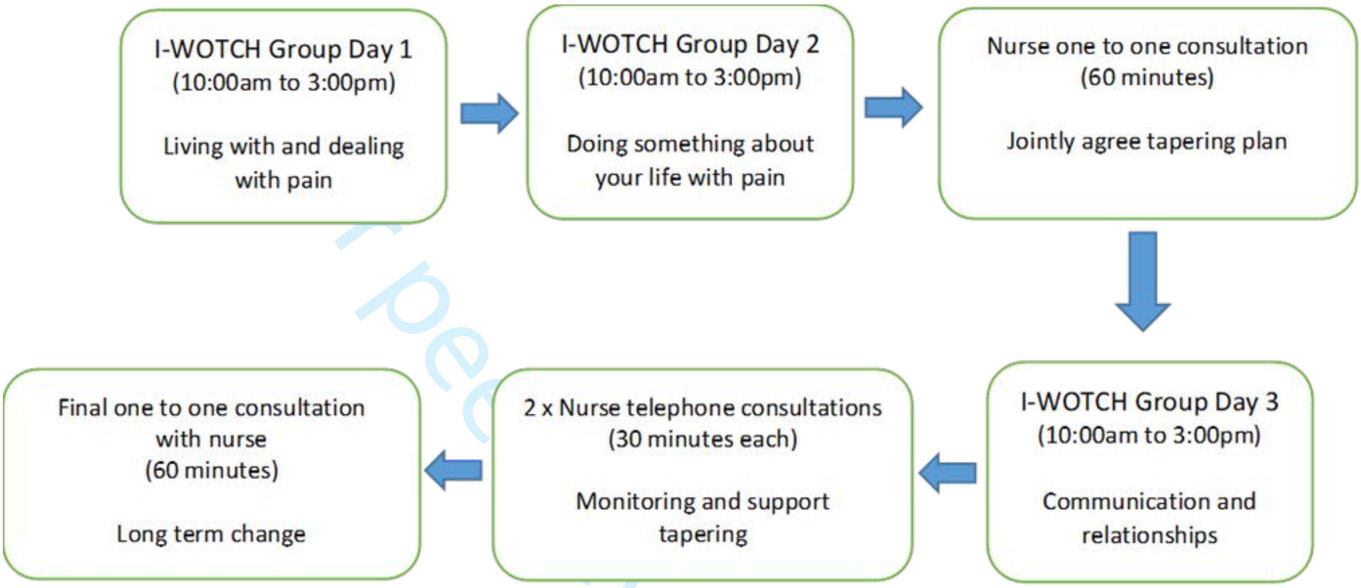

<u>One to one consultations</u>

The one-to-one sessions with a trained I-WOTCH nurse were based on a motivational interviewing model.(26) The aims of MI are to enhance behaviour change through a patient-centred framework, where the patient is able explore personal goals, ambivalence to change and reach self-actualisation in a supportive environment. We trained the I-WOTCH nurses on the five principles if motivational interviewing: Expressing empathy through reflective learning, Expressing empathy through reflective listening, developing discrepancy between participant goals or values (related to opioid tapering and pain management) and their current behaviour, avoiding argument and direct confrontation, adjusting to client resistance to reducing opioid reduction rather than opposing it directly and supporting self-efficacy and optimism. The one-to-one consultations included a review of medication, reflection on the

For peer review only - http://bmjopen.bmj.com/site/about/guidelines.xhtml

opioid education and group session where case studies and information were presented and exploring any challenges to opioid tapering such as concerns about withdrawal. Nurses were also trained to calculate total opioid daily dose and how to use that to produce a tapering regime according to the I-WOTCH study protocol. Although MI has been widely applied in substance misuse there are limited data available for its use in opioid cessation for people with chronic non cancer pain. A 2020 pilot study testing motivational interviewing opioid tapering in post joint arthroplasty surgery found a 62% increase in the rate of return to baseline opioid use after surgery (HR 1.62; 95%CI 1.06–2.46; p = 0.03).(27) Opioid tapering conversations maybe challenging and each participant will bring their own experiences and motivation to change, however by using MI as a tool we encouraged I-WOTCH facilitators to support participants in their tapering journey.(28)

One to one tapering – App

We adopted an opioid tapering regimen based on the Mayo Clinic experience as it provided some evidence to support the notion that slow tapering is unlikely to be associated with severe withdrawal symptoms and therefore likely to facilitate adherence.(29) This consisted of a 10% reduction of the original total daily dose every 7 days until a 30% of the original daily dose is reached. This is followed by a weekly decrease by 10% of the remaining dose. The 10% may be rounded up to suit prescribing. For the calculation of equianalgesic doses we used the tables from the Faculty of Pain Medicine.(30) In order to ensure standardisation of tapering methodology across sites and various opioid preparations the team developed a tapering App for use by the I-WOTCH trained nurses across sites. The I-WOTCH tapering App was developed by JN and SE working with the CTU programming team (HA, CM and AW) and provided to the nurses on a handheld tablet. The I-WOTCH tapering App was based on a mathematical algorithm applying the Mayo clinic regime while accounting for UK commercial preparations. Nurses used the App to generate a participant specific tapering plan, which was

For peer review only - http://bmjopen.bmj.com/site/about/guidelines.xhtml

automatically synchronised to the CTU team where it was printed and posted to the participant for information and GP for prescribing.

The I-WOTCH trained nurse entered the total daily dose of the participant-specific opioid preparation into the home screen of the App (e.g. 60mg oxycodone/day). The App algorithm then calculated 10% of the total daily dose and rounded this up or down to suit prescribing. All tablet, capsule or patch denominations of all opioid preparations were tabulated and added to the App to ensure the algorithm not only produced a 10% per week tapering regime but also a recommended various prescribing methods (e.g., oxycodone 35 mg could be prescribed as 30 and 5mg or 20,10 and 5mg tablets or 10,10,10 and 5mg tablets).

For patch preparations we advised participants to taper using their original opioid if 10% was not achievable (e.g., 12mcg of fentanyl being the smallest step down), the app algorithm was adjusted to recommend a 20% taper over a two-week period. Lowest dosage patch preparations were finally converted to slow release morphine equianalgesic doses and tapered accordingly.

My Opioid manager

The My Opioid Manager™ Book and App is the output of a project of Toronto Rehabilitation Institute, University Health Network. In 2010, Dr. Andrea Furlan, a Physician and Scientist at Toronto Rehabilitation Institute, developed a tool for physicians prescribing opioids for patients with chronic non-cancer pain. In 2012, the Opioid Manager™ was converted to an App for smartphones and tablets. The My Opioid Manager Book (and App) is intended to complement the Opioid Manager™ by providing the same information in a format that can be used by people with chronic pain who are on opioids, or by people who are not on opioids but who might be considering this option to help manage their chronic pain. The goal of My Opioid Manager is preparing the patient for upcoming consultations with their healthcare provider. Some of the topics discussed include: understanding the causes of various types of pains, uses

For peer review only - http://bmjopen.bmj.com/site/about/guidelines.xhtml

of opioids and the side effects and risks, managing pain by tracking opioid trials, and tips on using opioids. For this study we Anglicised the content in terms of language used as well as name of medication brands and pictures to be more representative of the UK population.

Venue for delivering the intervention

Where possible the I-WOTCH intervention is delivered in the community. Factors to consider when booking a venue include, access to building, parking and public transport links, a room to accommodate participants and facilitators with chairs and equipment, stairs, lifts, kitchen facilities and room for equipment such as flipchart, laptop screen, speakers and internet access.

I-WOTCH facilitator Training

The delivery–receipt–enactment chain of the I-WOTCH intervention provided a framework for training of facilitators and defining dosage received for participants to promote behaviour change (opioid tapering).(31) The I-WOTCH training included two full days for all facilitators (clinical and lay facilitators) and an additional day for clinical facilitators only, to learn the clinical aspects of tapering, opioid specific education, generating tapering plans and motivational interviewing for the one to one consultations. The design of the training package and implementation was adapted to Kolb's experiential learning cycle (training, experience and reflective observation).(32) The training days gave all facilitators exposure to the different components of the intervention through education and use of case studies. Trainers were given copies of the I-WOTCH manual and all participant intervention materials. Throughout the training days facilitators had the opportunity to ask questions and get clarity on any of the topics being covered. At the end of the training a short assessment was completed by each facilitator. If any of the facilitators scored below 70% they were then contacted by phone to go over any areas needing further explanation and offered further training if needed.

For peer review only - http://bmjopen.bmj.com/site/about/guidelines.xhtml

Discussion

We have used a methodical approach to developing an intervention to help people taking opioids for chronic pain to reduce opioid intake. This has been based on underpinning theory, the best available empirical evidence, and consultation with lay people. It has been piloted in and adapted in light of feedback. The I-WOTCH intervention has the potential to both help people reduce their opioid use and improve their overall quality of life. The I-WOTCH intervention is now being tested in the I-WOTCH trial. The trial protocol is published elsewhere.

Conclusion

We have designed an opioid reduction intervention package suitable for testing in a randomised controlled trial.

Collaborators I-WOTCH team:

Sharisse Alleyne, University of Warwick, Charles Abraham, Deakin University, Shyam Balasubramanian, Department of Anaesthesia & Pain Medicine University Hospitals Coventry and Warwickshire NHS Trust, Lauren Betteley, University of Warwick, Katie Booth, University of Warwick, Kirstie Haywood, University of Warwick, Sheeja Manchira Krishnan, The University of York, Ranjit Lall, University of Warwick. Andrea Manca, The University of York. Dipesh Mistry, University of Warwick, Vivien P Nichols, University of Warwick, Emma Padfield, University of Warwick, Anisur Rahman, Kate Seers, University of Warwick, University College London, Cynthia Paola Iglesis Urrutia,.

Contributors

All authors read and approved the manuscript. HS and SE are Co-Chief Investigators and oversee the running of the study. All named authors contributed to the design and/or delivery of the I-WOTCH intervention. HS, SE, JS and DC were involved in the design of the I-WOTCH intervention and design and delivery of facilitator training. CBT contributed to the

For peer review only - http://bmjopen.bmj.com/site/about/guidelines.xhtml

design and delivery of the I-WOTCH intervention, providing feedback on all materials and a trained facilitator. ADF developed My Opioid Manager, the content was anglicised for this study and also contributed to the design of the overall I-WOTCH intervention. SE, JN HA, CM and AW developed the I-WOTCH Opioid tapering App. MU, NYKT and SJCT contributed to the design of the intervention, training manuals and to this manuscript.

Funding

This project has received funding from the National Institute for Health Research (NIHR), Health Technology Assessment (HTA) (project number 14/224/04). The views and opinions expressed therein are those of the authors and do not necessarily reflect those of the HTA, NIHR, NHS or the Department of Health.

Ethical Approval

Ethics approval was given by Yorkshire & The Humber - South Yorkshire Research Ethics Committee on September 13th, 2016 **16/YH/0325**.

Competing interests

SE is the Chair of the specialised pain CRG at NHS England, he is Chief investigator and principal investigator of a number of NIHR and Industry funded trials, he has received personal fees from Medtronic Ltd, Mainstay Medical, Boston Scientific Corp for consultancy work. His department has received research funding from the National Institute of Health Research, Medtronic Ltd and Boston Scientific Corp. HS is director of Health Psychology Services Ltd, providing psychological services for a range of health related conditions. NT received grant funding as PI and CoI from NIHR for other projects

MU is chief investigator or co-investigator on multiple previous and current research grants from the UK National Institute for Health Research, Arthritis Research UK and is a co-investigator on grants funded by the Australian NHMRC. He was an NIHR Senior Investigator until March 2021. He has received travel expenses for speaking at conferences from the

For peer review only - http://bmjopen.bmj.com/site/about/guidelines.xhtml

professional organisations hosting the conferences. He is a director and shareholder of Clinvivo Ltd that provides electronic data collection for health services research. He is part of an academic partnership with Serco Ltd, funded by the European Social Fund, related to return to work initiatives. He receives some salary support from University Hospitals Coventry and Warwickshire He is a co-investigator on three NIHR funded studies receiving additional support from Stryker Ltd. He has accepted honoraria for teaching/lecturing from consortium for advanced research training in Africa. Until March 2020 he was an editor of the NIHR journal series, and a member of the NIHR Journal Editors Group, for which he received a fee. AF is author of the My Opioid Manager book and App distributed in iTunes and Google Play. Both book and app are free of charge. She is author of the Opioid Manager App, a paid app distributed only in iTunes for healthcare professionals. The app is owned by UHN, the hospital where AF works. AF does not get any financial benefit from the sales of the app. AF has a monetized YouTube channel since January 2021 that contains some videos about opioids and opioid tapering. Since April 2021, AF has a unrestricted educational grant to maintain an online self-assessment opioid course for healthcare professionals in Canada. The funding is provided by the Canadian Generics Pharmaceutical Association (CGPA). The funding organization has no role in the preparation, approval, recruitment of participants, or data analysis of the course content. Responsibility for the course content is solely that of the authors.

Acknowledgments

We would like to thank Ms Sally Brown for her valuable input in the development of the intervention. We would also like to thank all of our PPI volunteers and the Clinical Research Network (North East and Cumbria) for hosting the events which provided valuable insight to the structure and content of the intervention. We would like to thank our clinical and lay facilitators who attended training, delivered the intervention and provided valuable feedback. We would like to thank all participants involved in the pilot stages of the intervention. We

For peer review only - http://bmjopen.bmj.com/site/about/guidelines.xhtml

would also like to thank Dr Alison Hipwell for her contribution to the intervention development and Dr Celia Bernstein and Dr Jodie Westhead for their contribution to the I-WOTCH study.

References

1. Vos T, Abajobir AA, Abate KH, Abbafati C, Abbas KM, Abd-Allah F, Abdulkader RS, Abdulle AM, Abebo TA, Abera SF, Aboyans V. Global, regional, and national incidence, prevalence, and years lived with disability for 328 diseases and injuries for 195 countries, 1990–2016: a systematic analysis for the Global Burden of Disease Study 2016. The Lancet. 2017 Sep 16;390(10100):1211-59.

2. Bridges S. Health Survey for England 2011, Volume 1, Health , social care and lifestyles. HSCIC; 2012. p. 291-306.

3. International Association for the Study of Pain. Chronic Pain has arrived in the ICD-11 [Internet]. Washington: International Association for the study of Pain: 2019 Jan 17 [cited 2021 April 30]. Available from https://www.iasp pain.org/PublicationsNews/NewsDetail.aspx?ItemNumber=8340.

4. Mathieson S, Maher CG, Ferreira GE, Hamilton M, Jansen J, McLachlan AJ, Underwood M, Lin CW. Deprescribing Opioids in Chronic Non-cancer Pain: Systematic Review of Randomised Trials. Drugs. 2020 Jul 31:1-4.

5. Openprecribing, [Cited May 2021] Abailable from https://openprescribing.net/analyse/#org=stp&numIds=4.7.2&denom=nothing&selectedTab=chart

For peer review only - http://bmjopen.bmj.com/site/about/guidelines.xhtml

6. Baldini A, Von Korff M, Lin EH. A review of potential adverse effects of long-term opioid therapy: a practitioner's guide. The primary care companion to CNS disorders. 2012;14(3).).

7. Chou R, Turner JA, Devine EB, Hansen RN, Sullivan SD, Blazina I, Dana T, Bougatsos C, Deyo RA Ann Intern Med. 2015 Feb 17;162(4):276-86. doi: 10.7326/M14-2559. Review. PMID:25581257

8. Sandhu H, Underwood M, Furlan AD, Noyes J, Eldabe S. What interventions are effective to taper opioids in patients with chronic pain?. bmj. 2018 Sep 27;362.

9. Sandhu H, Underwood M, Furlan AD, Noyes J, Eldabe S. What interventions are effective to taper opioids in patients with chronic pain?. bmj. 2018 Sep 27;362.

10. O'Cathain A, Croot L, Sworn K, Duncan E, Rousseau N, Turner K, Yardley L, Hoddinott P. Taxonomy of approaches to developing interventions to improve health: a systematic methods overview. Pilot and feasibility studies. 2019 Dec;5(1):1-27.).

11. Craig P, Dieppe P, Macintyre S, Michie S, Nazareth I, Petticrew M. Developing and evaluating complex interventions: the new Medical Research Council guidance. BMJ (Clinical research ed). 2008;337:a1655.]

12. Carnes D, Homer K, Underwood M, Pincus T, Rahman A, Taylor S.J.C. Pain management for chronic musculoskeletal conditions: the development of an evidence based and theory informed pain self-management course. BMJ Open. 2013; 3:e003534.doi:10.1136

13. Univeristy of Warwick. UNTRAP Leaflet [Internet]. Coventry: Univeristy of Warwick [Cited May 2021]. Available from https://warwick.ac.uk/fac/cross_fac/healthatwarwick/untrap/untrapleaflet.pdf.

For peer review only - http://bmjopen.bmj.com/site/about/guidelines.xhtml

14. Turk DC, Wilson H, Swanson KS, Ebert M, Kerns R. The biopsychosocial model of pain and pain management. Cambridge: Cambridge University Press; 2011.

15. Abraham C, Michie S. A taxonomy of behavior change techniques used in interventions. Health Psychol 2008;27:379–87

16. Richards, Georgia C.a,*; Lluka, Lesley J.b; Smith, Maree T.c; Haslam, Catherined; Moore, Brendanc,e; O'Callaghan, Jamese; Strong, Jennyf Effects of long-term opioid analgesics on cognitive performance and plasma cytokine concentrations in patients with chronic low back pain: a cross-sectional pilot study, PAIN Reports: July/August 2018

17. Diamond A. Executive functions. Annual review of psychology. 2013 Jan 3;64:135-68.

18. Bandura A. Self-efficacy: toward a unifying theory of behavioral change. Psychological review. 1977 Mar;84(2):191.

19. Ajzen I, Fishbein M. Understanding attitudes and predicting social behavior. London: Pearson Education, 1980

20. Ajzen I, Fishbein M. Belief, attitude, intention and behaviour: an introduction to theory and research. Higher Education; 1976.

21. Bandura A. Social foundations of thought and action. Englewood Cliffs, NJ. 1986;1986(23-28).

For peer review only

22. Morley S, Shapiro DA, Biggs J. Developing a treatment manual for attention management in chronic pain. Cogn Behav Ther 2004;33:1–11.

23. Kuhl J, Beckmann J, editors. Action control: From cognition to behavior. Springer Science & Business Media; 2012 Dec 6.

24. Hayes SC, Strosahl KD, Wilson KG. Acceptance and commitment therapy: an experiential approach to behavior change. New York: Guilford Press, 2004.

25. Carnes D, Homer K, Underwood M, Pincus T, Rahman A, Taylor SJ. Pain management for chronic musculoskeletal conditions: the development of an evidence-based and theory-informed pain self-management course. BMJ open. 2013 Nov 1;3(11).

26. Miller WR, Rollnick S. Motivational interviewing: Preparing to change addictive behaviour. New York: Guilford. 1991.

27. Hah JM, Trafton JA, Narasimhan B, Krishnamurthy P, Hilmoe H, Sharifzadeh Y, Huddleston JI, Amanatullah D, Maloney WJ, Goodman S, Carroll I. Efficacy of motivational-interviewing and guided opioid tapering support for patients undergoing orthopedic surgery (MI-Opioid Taper): A prospective, assessor-blind, randomized controlled pilot trial. EClinicalMedicine. 2020 Nov 1;28:100596.).

28. Crawley A, Murphy L, Regier L, McKee N. Tapering opioids using motivational interviewing. Canadian Family Physician. 2018 Aug 1;64(8):584-7.

For peer review only - http://bmjopen.bmj.com/site/about/guidelines.xhtml

29. Berna C, Kulich RJ, Rathmell JP. Tapering long-term opioid therapy in chronic noncancer pain: evidence and recommendations for everyday practice. InMayo Clinic Proceedings 2015 Jun 1 (Vol. 90, No. 6, pp. 828-842). Elsevier.

30. Faculty of Pain Medicine of Royal College of Anaesthetists. Dose equivalents and changing opioids [Internet]. London: Faculty of Pain Medicine of Royal College of Anaesthetists. Available from: https://fpm.ac.uk/opioids-aware-structured-approach-opioid-prescribing/dose-equivalents-and-changing-opioids

31. Sandhu HK, Abraham C, Alleyne S, Balasubramanian S, Betteley L, Booth K, Carnes D, Furlan AD, Haywood K, Urrutia CP, Lall R. Testing a support programme for opioid reduction for people with chronic non-malignant pain: the I-WOTCH randomised controlled trial protocol. BMJ open. 2019 Aug 1;9(8):e028937.

32. Kolb DA. Experience as the source of learning and development. Upper Sadle River: Prentice Hall. 1984.

For peer review only

Figure 1: Stages of I-WOTCH intervention development

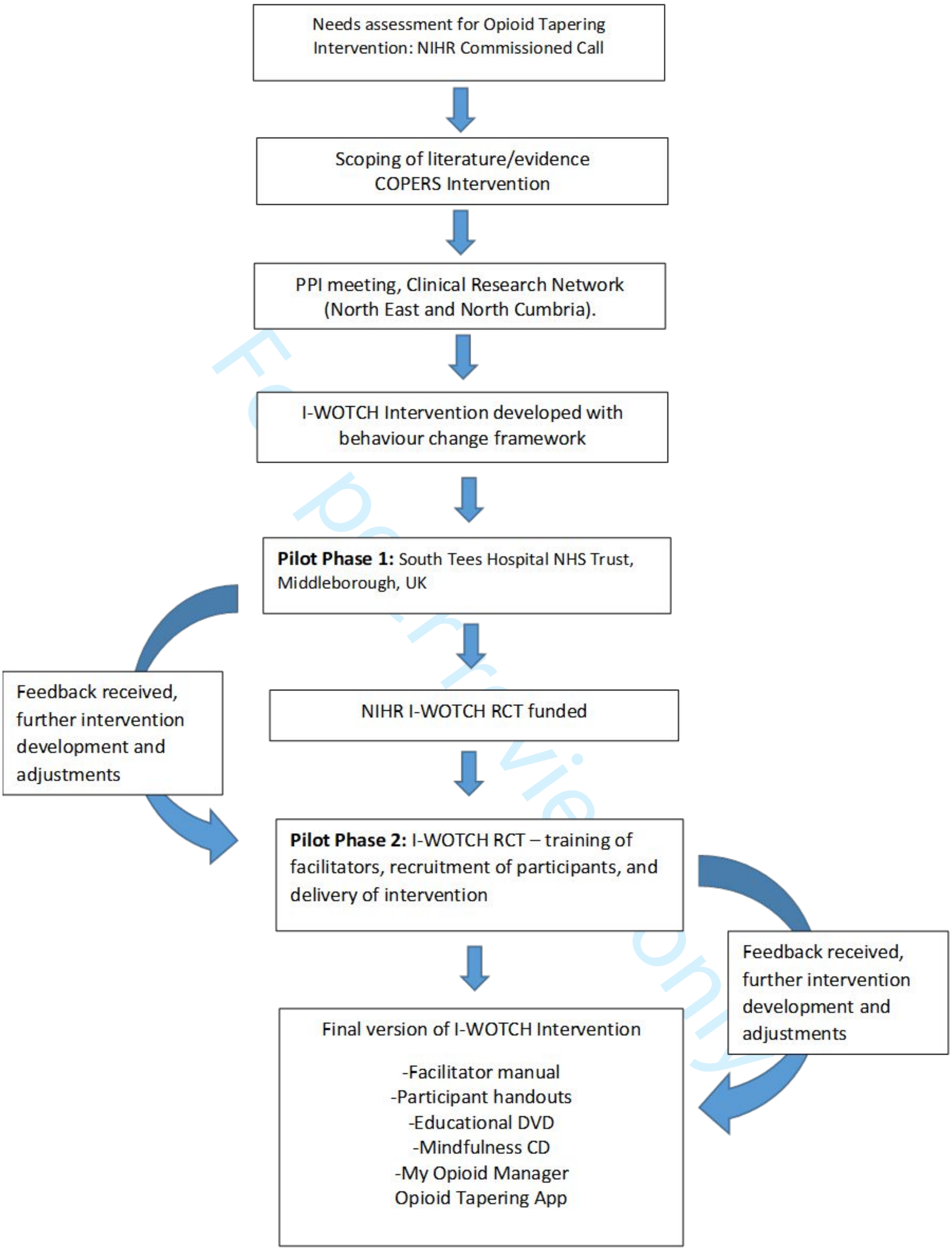

Legend:
COPERS - coping with persistent pain, effectiveness research into self-management (12)
I-WOTCH- Improving the Wellbeing of Opioid Treated Chronic Pain
NIHR – National Institute of Health Research
PPI - Patient and Public Involvement
RCT – Randomised Controlled Trial

For peer review only - http://bmjopen.bmj.com/site/about/guidelines.xhtml

Figure 2: Reducing Opioids for patients with chronic non cancer pain

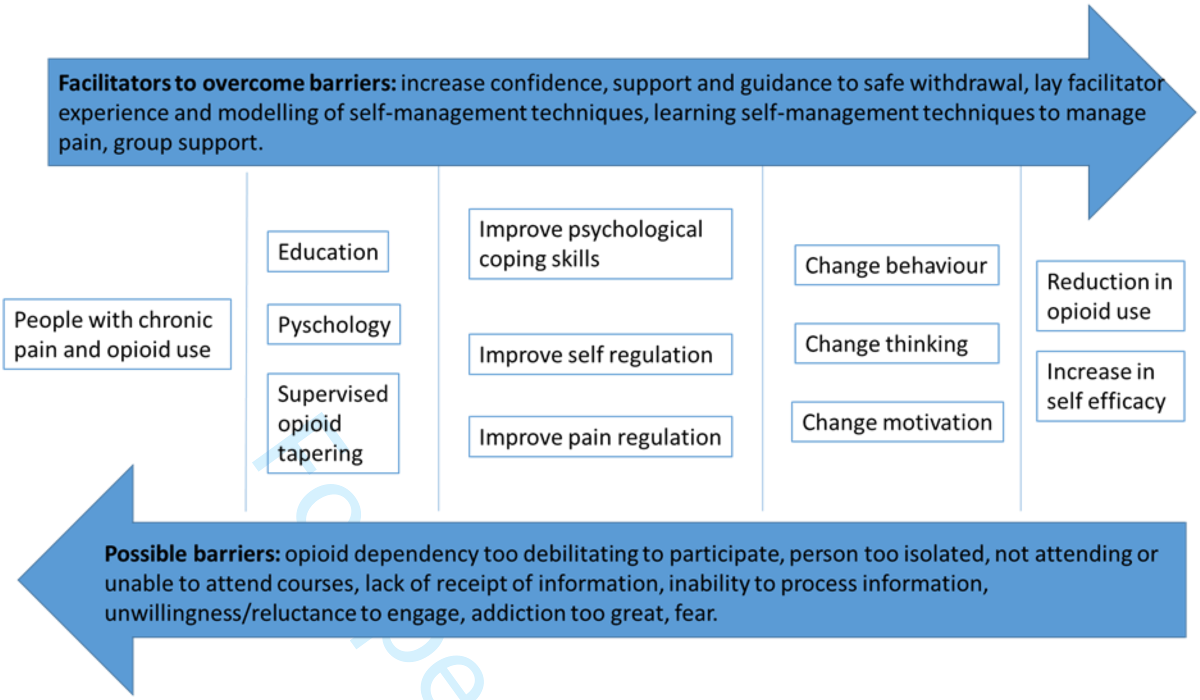

For peer review only

BMJ Open

The development and testing of an opioid tapering self-management intervention for chronic pain – I-WOTCH

Journal:	*BMJ Open*
Manuscript ID	bmjopen-2021-053725.R1
Article Type:	Original research
Date Submitted by the Author:	08-Dec-2021
Complete List of Authors:	Sandhu, Harbinder; University of Warwick Warwick Medical School, Clinical Trials Unit<br>Shaw, Jane; James Cook University Hospital<br>Carnes, Dawn; Queen Mary University of London Barts and The London School of Medicine and Dentistry, Institute of Population Health Sciences<br>Furlan, Andrea; Toronto Rehabilitation Institute; University of Toronto Faculty of Medicine<br>Tysall, Colin; University of Warwick, UNTRAP; Coventry University, SUCE<br>Adjei, Henry; University of Warwick Warwick Medical School, Warwick Clinical Trials Unit<br>Muthiah, Chockalingam; University of Warwick, Warwick Clinical Trials Unit<br>Noyes, Jennifer; James Cook University Hospital, Department of Pain Medicine<br>Tang, Nicole; University of Warwick, Psychology<br>Taylor, Stephanie; Queen Mary University of London Barts and The London School of Medicine and Dentistry, Wolfson Institute of Population Health<br>Underwood, Martin; University of Warwick Warwick Medical School, Warwick Clinical Trials Unit<br>Willis, Adrian; University of Warwick, Warwick Clinical Trials Unit<br>Eldabe, Sam; James Cook University Hospital, Department of Pain Medicine
**Primary Subject Heading**:	Medical management
Secondary Subject Heading:	Patient-centred medicine, Pharmacology and therapeutics, Rehabilitation medicine
Keywords:	Pain management < ANAESTHETICS, Rehabilitation medicine < INTERNAL MEDICINE, MEDICAL EDUCATION & TRAINING, PAIN MANAGEMENT, PRIMARY CARE, Clinical trials < THERAPEUTICS

SCHOLARONE™
Manuscripts

I, the Submitting Author has the right to grant and does grant on behalf of all authors of the Work (as defined in the below author licence), an exclusive licence and/or a non-exclusive licence for contributions from authors who are: i) UK Crown employees; ii) where BMJ has agreed a CC-BY licence shall apply, and/or iii) in accordance with the terms applicable for US Federal Government officers or employees acting as part of their official duties; on a worldwide, perpetual, irrevocable, royalty-free basis to BMJ Publishing Group Ltd ("BMJ") its licensees and where the relevant Journal is co-owned by BMJ to the co-owners of the Journal, to publish the Work in this journal and any other BMJ products and to exploit all rights, as set out in our licence.

The Submitting Author accepts and understands that any supply made under these terms is made by BMJ to the Submitting Author unless you are acting as an employee on behalf of your employer or a postgraduate student of an affiliated institution which is paying any applicable article publishing charge ("APC") for Open Access articles. Where the Submitting Author wishes to make the Work available on an Open Access basis (and intends to pay the relevant APC), the terms of reuse of such Open Access shall be governed by a Creative Commons licence – details of these licences and which Creative Commons licence will apply to this Work are set out in our licence referred to above.

Other than as permitted in any relevant BMJ Author's Self Archiving Policies, I confirm this Work has not been accepted for publication elsewhere, is not being considered for publication elsewhere and does not duplicate material already published. I confirm all authors consent to publication of this Work and authorise the granting of this licence.

For peer review only

The development and testing of an opioid tapering self-management intervention for chronic pain: I-WOTCH

Harbinder Kaur Sandhu, Jane Shaw, Dawn Carnes, Andrea Dompieri Furlan, Colin Bernard Tysall, Henry Adjei, Chockalingam Muthiah, Jennifer Noyes, Nicole K Y Tang , Stephanie JC Taylor, Martin Underwood, Adrian Willis, Sam Eldabe, On behalf of the I-WOTCH Team

Harbinder Kaur Sandhu, Warwick Clinical Trials Unit, Warwick Medical School, University of Warwick, Coventry CV4 7AL, UK

Harbinder.K.Sandhu@warwick.ac.uk

Jane Shaw, The James Cook University Hospital, Middlesbrough, TS4 3BW, UK

Jane@janeshaw.net

Dawn Carnes PhD, Institute of Population Health Sciences, Barts and The London School of Medicine and Dentistry, Queen Mary University of London, UK

d.carnes@qmul.ac.uk

Andrea Dompieri Furlan, Toronto Rehabilitation Institute, University Health Network, Department of Medicine, University of Toronto, Institute for Work & Health, Toronto Canada

Andrea.furlan@uhn.ca

Colin Bernard Tysall, UNTRAP Warwick University, SUCE Coventry University, UK

colin@tysall.co.uk

For peer review only - http://bmjopen.bmj.com/site/about/guidelines.xhtml

Henry Adjei, Warwick Clinical Trials Unit, Warwick Medical School, University of Warwick, Coventry CV4 7AL, UK

H.Adjei@warwick.ac.uk

Chockalingam Muthiah, Warwick Clinical Trials Unit, Warwick Medical School, University of Warwick, Coventry CV4 7AL, UK

c.muthiah@warwick.ac.uk

Jennifer Noyes, Department of Pain Medicine, Murray Building, The James Cook University Hospital, Middlesbrough, TS4 3BW, UK

j.noyes@nhs.net

Nicole K Y Tang, Department of Psychology, University of Warwick, Coventry CV4 7AL, UK

N.Tang@warwick.ac.uk

Stephanie JC Taylor, Wolfson Institute of Population Health, Barts and The London School of Medicine and Dentistry, Queen Mary University of London, UK

s.j.c.taylor@qmul.ac.uk

Martin Underwood, Warwick Clinical Trials Unit, Warwick Medical School, University of Warwick, Coventry CV4 7AL, UK and University Hospitals Coventry and Warwickshire, Clifford Bridge Road Coventry CV2 2DX, UK

M.Underwood@warwick.ac.uk

For peer review only

For peer review only - http://bmjopen.bmj.com/site/about/guidelines.xhtml

Adrian Willis, Warwick Clinical Trials Unit, Warwick Medical School, University of Warwick, Coventry CV4 7AL, UK

Adrian.Willis@warwick.ac.uk

Sam Eldabe, Department of Pain Medicine, Murray Building, The James Cook University Hospital, Middlesbrough, TS4 3BW, UK

seldabe@nhs.net

Corresponding Author: Harbinder Kaur Sandhu, Warwick Clinical Trials Unit, Warwick Medical School, University of Warwick, Coventry CV4 7AL, UK

Harbinder.K.Sandhu@warwick.ac.uk

Word count: 3396

For peer review only

Abstract

Objectives: To describe the design, development and pilot of a multi-component intervention aimed at supporting withdrawal of opioids for people with chronic non-malignant pain for future evaluation in the Improving the Wellbeing of Opioid Treated CHronic pain (I-WOTCH) randomised controlled trial.

Design: The I-WOTCH intervention draws on previous literature and co-creation with stakeholders (patient and public involvement). Intervention mapping and development activities of Behaviour Change Taxonomy are described.

Setting: The intervention development was conducted by a multidisciplinary team with clinical, academic and service user perspectives. The team had expertise in the development and testing

For peer review only - http://bmjopen.bmj.com/site/about/guidelines.xhtml

of complex health behaviour interventions, opioid tapering and pain management in primary and secondary care, I.T programming, and software development - to develop an opioid tapering App.

Participants: The I-WOTCH trial participants are adults (18 years and over) with chronic non-malignant pain using strong opioids for at least three months and on most days in the preceding month.

Outcomes: A multi-component self-management support package to help people using opioids for chronic non-malignant pain reduce opioid use.

Interventions and Results: Receiving information on the impact of long-term opioid use, and potential adverse effects were highlighted as important facilitators in making the decision to reduce opioids. Case studies of those who have successfully stopped taking opioids were also favoured as a facilitator to reduce opioid use. Barriers included the need for a "trade-off to fill the deficit of the effect of the drug". The final I-WOTCH intervention consists of an 8 to 10 week programme incorporating: education; problem solving; motivation; group and one to one tailored planning; reflection and monitoring. A detailed facilitator manual was developed to promote consistent delivery of the intervention across the UK.

Conclusions: We describe the development of an opioid reduction intervention package suitable for testing in the I-WOTCH randomised controlled trial.

Trial Registration: ISRCTN49470934

Article summary

Strengths and limitations:

For peer review only - http://bmjopen.bmj.com/site/about/guidelines.xhtml

1. The I-WOTCH Intervention draws on psychological and behaviour change frameworks.

2. The I-WOTCH intervention was co-created with key stakeholders including patient and public involvement (those with chronic-non-malignant pain and experience of opioid use and/or tapering).

3. The pilot phases and feasibility testing gave valuable feedback and changes were made to the intervention accordingly.

4. At the time of designing the intervention there was limited previous work and information to inform content of the intervention.

Funding Statement:

This project has received funding from the National Institute for Health Research (NIHR), Health Technology Assessment (HTA) (project number 14/224/04). SJCT supported by the National Institute for Health Research ARC North Thames. The views and opinions expressed therein are those of the authors and do not necessarily reflect those of the HTA, NIHR, NHS or the Department of Health.

Competing Interests

SE is the Chair of the specialised pain CRG at NHS England, he is Chief investigator and principal investigator of a number of NIHR and Industry funded trials, he has received personal fees from Medtronic Ltd, Mainstay Medical, Boston Scientific Corp for consultancy work. His department has received research funding from the National Institute of Health Research, Medtronic Ltd and Boston Scientific Corp. HS is director of Health Psychology Services Ltd, providing psychological services for a range of health related conditions. NKYT is chief

For peer review only - http://bmjopen.bmj.com/site/about/guidelines.xhtml

For peer review only

investigator or co-investigator of other chronic pain related projects funded by the NIHR, MRC, Warwick-Wellcome Translational Partnership.

MU is chief investigator or co-investigator on multiple previous and current research grants from the UK National Institute for Health Research, Arthritis Research UK and is a co-investigator on grants funded by the Australian NHMRC. He was an NIHR Senior Investigator until March 2021. He has received travel expenses for speaking at conferences from the professional organisations hosting the conferences. He is a director and shareholder of Clinvivo Ltd that provides electronic data collection for health services research. He is part of an academic partnership with Serco Ltd, funded by the European Social Fund, related to return to work initiatives. He receives some salary support from University Hospitals Coventry and Warwickshire He is a co-investigator on three NIHR funded studies receiving additional support from Stryker Ltd. He has accepted honoraria for teaching/lecturing from consortium for advanced research training in Africa. Until March 2020 he was an editor of the NIHR journal series, and a member of the NIHR Journal Editors Group, for which he received a fee.

AF is author of the My Opioid Manager book and App distributed in iTunes and Google Play. Both book and app are free of charge. She is author of the Opioid Manager App, a paid app distributed only in iTunes for healthcare professionals. The app is owned by UHN, the hospital where AF works. AF does not get any financial benefit from the sales of the app. AF has a monetized YouTube channel since January 2021 that contains some videos about opioids and opioid tapering. Since April 2021, AF has an unrestricted educational grant to maintain an online self-assessment opioid course for healthcare professionals in Canada. The funding is provided by the Canadian Generics Pharmaceutical Association (CGPA). The funding organization has no role in the preparation, approval, recruitment of participants, or data analysis of the course content. Responsibility for the course content is solely that of the authors.

For peer review only - http://bmjopen.bmj.com/site/about/guidelines.xhtml

SJCT is chief investigator or co-investigator on multiple previous and current research grants from the UK National Institute for Health Research.

Article Summary: Strengths and limitations

Key words: Opioid withdrawal, medication reduction, behaviour change, chronic non malignant pain, self-management, intervention development

For peer review only

Introduction

Pain, and pain related disorders, continue to be the leading cause of disability and disease burden globally (1), with low back pain making the largest contribution to years lived with disability. In England at least eight million people (15% of the population) have moderate to severe persistent (chronic) pain (2) defined as *pain that lasts or recurs for more than three months.*(3) Over the past few decades, there has been a global epidemic of opioid prescribing for chronic non-malignant pain. A 2020 systematic review found that 30 percent of people with chronic non-malignant pain are prescribed opioid medication and, globally, this has steadily increased until recently with time. (4) In the UK prescribing rates have decreased slightly over recent years, however the number of prescriptions remains high.(5) Long term use of opioids leads tolerance and loss of effective pain relief. Adverse consequences include opioid induced hyperalgesia, endocrine disorders and hypogonadism, drowsiness, a high risk of dependency, opioid use disorder, sleep apnoea, sexual dysfunction, immune suppression, falls leading to increased fractures (particularly a risk in the elderly population) and increased risk for overdose and death.(6) There are limited strategies to help with risk mitigation and interventions to help people with chronic non-malignant pain withdraw from opioids.(7) A 2020 systematic review found ten randomised controlled trials (n=835) of patient-focused opioid de-prescribing interventions targeting people with chronic non-malignant pain. These included: dose

For peer review only - http://bmjopen.bmj.com/site/about/guidelines.xhtml

reduction protocols (weekly reduction of 10 percent); opioid replacement including (buprenorphine, morphine sulphate or oxycodone hydrochloride or varenicline; non-pharmacological therapies including mindfulness (vs active control or support group); therapeutic interactive voice response programme (vs usual care); meditation; cognitive behavioural therapy (vs usual care); and electroacupuncture (vs sham). The primary outcome was mean reduction of daily dose in morphine milligram equivalents). Only one study reported a statistically significant difference in the daily dose between groups in favour of the intervention (a study using a dose tapering protocol) (Mean Difference -27.9 MME/day, 95%CI -41.1 to -14.7).(8) None of these interventions reported increases in opioid cessation in the intervention groups. Overall, the authors were unable to recommend any particular deprescribing strategy due to the small number of studies and heterogeneity of the data.(4)

Current recommendations on opioid tapering are based on best practice and guidelines which need to be supported by further evidence.(9) Here we describe the development of a multi-component opioid tapering programme (incorporating group and one to one sessions) as part of the I-WOTCH study (Improving the Wellbeing of Opioid Treated Chronic Pain), funded by the National Institute of Health Research [14/224/04]. The I-WOTCH study protocol has been published previously.(10)

Methods

The I-WOTCH intervention was developed in collaboration with the target population (those with chronic non-malignant pain and experience of opioid use). It employed theory and evidence based implementation (with a view to implementation in the real world should it be effective) and included digital technologies to generate opioid tapering plans.(11) The Medical Research Council Framework (12) for designing complex interventions, evidence based

For peer review only - http://bmjopen.bmj.com/site/about/guidelines.xhtml

interventions (13) and core theoretical principles was used to inform the design of content, structure, and delivery of the intervention.

Key stages of the intervention development, figure 1. Adjustment and adaptation to the intervention were implemented in-line with feedback received from stakeholders (service users, clinicians and facilitators delivering the I-WOTCH intervention).

Aims and objectives of the I-WOTCH intervention

In line with the overall study, the aims of the I-WOTCH intervention were:

1) To reduce opioid and healthcare use for people with chronic, non-malignant pain
2) To increase study participants' self-efficacy (confidence) to reduce opioid medication and implement self-management strategies of pain.
3) To improve quality of life and help people live better with pain.

Objectives:

1) To provide education using a range of teaching methods; group discussion, problem solving, experiential learning and case studies.
2) To provide an environment which enhances motivation to reduce opioid use through group cohesion and one to one support.
3) To provide an overall cost-effective intervention to be implemented in healthcare services.

Defining the aims and objectives enabled us to consider what we wanted to achieve, how and for what purpose. In addition, we were aware of potential facilitators and barriers that could influence engagement with the intervention and the procedures of the trial. Figure 2 shows the direction of travel we were aiming for and what we needed to consider when designing the detail of intervention and mechanism of behaviour change.

Patient and public involvement

For peer review only - http://bmjopen.bmj.com/site/about/guidelines.xhtml

During the development stages of I-WOTCH, we held two PPI meetings with the Clinical Research Network (North East and Cumbria) at The James Cook University Hospital (South Tees Hospitals NHS Foundation Trust). A total of nineteen volunteer participants (people with chronic pain and experience of opioid therapy and/or opioid tapering) attended. Discussions were facilitated by members of the study team (HS, DC, JS and SE) and included, intervention structure and design, content (topics to cover which would potentially increase motivation and confidence to taper opioids), length of programme, where the intervention should be delivered, support during opioid tapering (including frequency of contact with healthcare professionals) and delivery of the intervention (who should deliver the intervention) (Table 1). In addition to this, two lay advisors who were part of the I-WOTCH study recruited via Universities/User Teaching, (14) gave considerable input into the design of, and training to deliver, the intervention.

Table 1: Feedback from PPI Informing Intervention Development

**Discussion topic**	**Feedback informing intervention development**
Behaviour change	Agreed aims should be a reduction in opioid consumption and engagement in the I-WOTCH programme.<br><br>Behaviour change needs to be accepted before opioid reduction can occur.
Understanding motivation to change behaviour	Changing medication and reducing medication can be motivated by:<br><br>i) a trade-off to fill the deficit of the effect of the drug (something else needed that is as effective as the drug they would lose)<br>ii) reduction in side effects<br><br>Use of case studies of people who had successfully stopped taking opioids would be useful.
Content and topics to be covered	The intervention would benefit from being informative (opioid education, especially long-term consequences, pros and cons of opioid use and managing withdrawal).<br><br>The following topics were recommended for inclusion:<br>• What is Pain

For peer review only - http://bmjopen.bmj.com/site/about/guidelines.xhtml

	• Acceptance – pain and learning to live better with pain<br>• Impact of pain – and integrate this information with taking medication (Opioids), why and how?<br>• The importance of hobbies and having a distraction to manage the pain<br>• Offer alternative non-pharmacological ways of coping, e.g. mindfulness and relaxation<br>• Incorporate movement<br>• Guidance on posture and exercise/activity<br>• Pacing – not over doing things
Dependency vs addiction	It was felt important to distinguish between dependency and addiction, as some were concerned about the stigma and labels attached to long term opioid use for chronic pain.
Delivery of I-WOTCH Intervention, who?	Feedback favoured the course to be delivered jointly by a HCP* and a lay facilitator (someone who had experience of long term pain and opioid use/tapering).
Structure of Intervention	Group and individual care approaches were valued.<br>Length of the proposed programme (3-day group sessions and ongoing one to one support) was supported.<br>The duration of intervention was not viewed as burdensome given that some had people who have experienced severe withdrawal symptoms, and therefore ongoing support over the 8 to 10 weeks is needed.<br><br>There was a consensus that a group-based format and group cohesion would be optimal because of the potential for social comparison, social validation and development of social support. Volunteers identified the impact of opioid use on enhanced day-to-day activities as important evaluation outcomes, including: work productivity, looking after children, and overall functioning.
Communication during study	Volunteers welcomed the idea of having a study website to give participants an opportunity to be updated about the study as a whole and progress.

*HCP Health care professional

Opioid Tapering and Behaviour Change

The target behaviour change was defined as the participants engaging in the I-WOTCH intervention, reducing participant opioid use, and implementing non-pharmacological strategies of pain management. The bio-psychosocial framework (15), Michie's taxonomy of behaviour change and the COM-B framework for behaviour change (Capability, Opportunity,

For peer review only - http://bmjopen.bmj.com/site/about/guidelines.xhtml

For peer review only

Motivation) were consulted.(16) Capability includes psychological capability (e.g., can patients engage in the necessary thought processes needed to engage in the tapering processes?) and physical capability (e.g., do participants have the capacity to engage in the tapering?). Psychological capability is broken down to cognitive functioning and executive functioning. To promote cognitive functioning (which includes a range of mental abilities such as learning, problem solving and attention) we produced handouts of material covered on each day the programme. This allowed opportunity for participants to recap over the core messages and information in their own time. We also included time for group reflection at the start of each session and summarising discussions at the end of each of the group days (with opportunities for questions). In addition to this we developed an educational DVD, a mindfulness CD and relaxation CD for each participant (at the time we developed the intervention DVDs and CDs were still in common use). By providing material to take home we were giving participants an opportunity to revisit and take in the information at their own pace.(17) Executive functioning includes the capacity to plan and think, explore challenges that may occur (e.g.,fear of withdrawal symptoms), stay focused on the goal (opioid reduction) and resist temptation.(18) In the I-WOTCH intervention we gave participants opportunity to set goals (through an educational session and support in generating goals related to opioid tapering and their general life). We also encouraged self-reflection to identify perceived barriers and facilitators to tapering and gave further guidance to overcome the perceived barriers in the tailored one to one support sessions with the clinical facilitator. Physical capability refers to whether the participants exposed to the I-WOTCH intervention felt they had the right skills to engage in the tapering of their opioids, this may include management of withdrawal, confidence and having structure and support in place. The I-WOTCH intervention was designed to help participants adapt and put into place lifestyle changes.

For peer review only - http://bmjopen.bmj.com/site/about/guidelines.xhtml

Opportunity is the second component of the COM-B model. For this we explored factors external to the individual that would promote opioid tapering. For example, physical opportunity which includes, costs of opioids and travel, access and availability, developing a tapering plan (clear and informative) and enhancing communication between the clinical facilitator and participant through motivational interviewing during the tapering processes. In relation to social opportunity, we referred to what other factors may impact the decision to taper such as stigma and cultural beliefs.

Motivation, this refers to both the cognitive motivation and emotional processes to energise and direct the behaviour change of opioid tapering. Reflective processes included exploring perceptions and meaning of chronic pain during the group sessions as well as beliefs about tapering, possible outcomes concerns and self-efficacy. There was opportunity to evaluate and be reflective during the group sessions as well as one to one support. Automatic processes refer to the emotional responses which may occur during the I-WOTCH intervention and these include anxiety, fear, stress, and low mood. All topics were covered in the group sessions including recognition of thoughts and emotions and management strategies.

Each component of the I-WOTCH intervention was informed and mapped on to behaviour change taxonomy's (BCTv1). The intervention also drew on psychological theories of self-efficacy (19), Theory of planned behaviour and reasoned action,(20, 21) social learning (22) and group based interventions,(23) cognitive behaviour-change, (24) motivational interviewing (25) and evidence based interventions for self-managing chronic pain (COPERS) (26) described in Table 2.

For peer review only - http://bmjopen.bmj.com/site/about/guidelines.xhtml

I-WOTCH Group based sessions day 1 (week 1)	Aims	Theoretical Underpinnings	Behaviour Change Taxonomy
Introductions, group work, aims	To allow participants to introduce themselves to the group, encourage participation in a safe and relaxed environment, explore expectations and discuss the I-WOTCH course aims	Social cognitive theory<br><br>Bio-psychosocial theory	Improve bonding and group cohesion.<br><br>Breaking barriers and encouraging self and social awareness
What Causes Pain? (Pain information)	To increase understanding about long-term pain	Biopsychosocial theory<br><br>Principles of self-efficacy and acceptance	Credible Source
Living With Pain (Opioid education I)	To increase understanding about use of opioids for long term pain and encourage participants to start questioning their own knowledge and beliefs about opioids and why they take them	Biopsychosocial theory<br><br>Theory of planned behaviour and reasoned action<br><br>Health Beliefs	Information about health consequences
Acceptance	To understand and start to accept pain, with a view to implementing self-management strategies as reduction of opioids occurs	Acceptance and Self-management of chronic pain	Goal setting<br><br>Commitment
Attention Control & Distraction	To learn how to focus the mind away from pain thoughts and use of opioids	Cognitive behaviour change<br><br>Self-management of chronic pain<br><br>Health beliefs	Distraction
Distraction activity – drawing	An opportunity to practise distraction	Cognitive behaviour change	Behavioural practice Distraction

Table 2: Behaviour change taxonomy and opioid tapering

For peer review only - http://bmjopen.bmj.com/site/about/guidelines.xhtml

	activity and socially interact with group informally	Social learning	
Good days, bad days - Pain, bearable or not?	To reinforce that pain is not just physiological, it is psychological, social and an emotional phenomenon	Biopsychosocial theory<br><br>Health beliefs	Information and antecedents<br><br>Information about health consequences<br><br>Re-attribution of behaviour
The pain cycle (including opioids) and breaking the pain cycle	To explain and identify unhelpful factors in the pain cycle and learn strategies to break the cycle	Biopsychosocial theory<br><br>Health beliefs	Behaviour Substitution (adding in other behaviours to break cycle)
Posture and movement	To promote body awareness, posture and muscle weakness (Managing pain without opioids)	Theory of planned behaviour and reasoned action	Guidelines on exercise, physical therapy principles<br><br>Mindfulness
Relaxation and breathing	To reduce muscle tension and introduce breathing as a relaxation technique	Cognitive behaviour change<br><br>Self-management of chronic pain	Behavioural practice<br><br>Distraction<br><br>Body changes
Summary of the day	To consolidate learning of the day and outline aims for group day 2.	Acceptance and principles of self-efficacy	Action planning<br><br>Verbal persuasion about capability
I-WOTCH group based Sessions Day 2 (week 2)	Aims	Theoretical Underpinnings	Behaviour Change Taxonomy
Reflections from day 1	To understand and empathise with the group	Social learning<br><br>Self-efficacy	Improve bonding and group cohesion, social cognitive theory
Stress-busting for Health: Action planning, problem solving, pacing, SMART goal setting	To help the participants logically and systematically identify problems, free think solutions, set achievable goals and create action plans, as a means of escaping the pain cycle	Cognitive behaviour change<br><br>Theory of planned behaviour and reasoned action	Goal setting<br><br>Comparative imagining of future outcomes<br><br>Reduce negative emotions<br><br>Problem solving
Withdrawal symptoms, Case	To discuss potential withdrawal symptoms	Health beliefs	Social comparison (drawing attention to

For peer review only - http://bmjopen.bmj.com/site/about/guidelines.xhtml

studies (Opioid education II)	that participants might experience if their taper is too quick	Social learning	others' performance to allow comparison with the person's own performance)<br>Credible source<br><br>Comparative imagining of future outcomes
Distraction activity – origami	To learn how to focus the mind away from pain thoughts and use of opioids	Cognitive behaviour change<br><br>Social learning	Behavioural practice<br><br>Distraction
Identifying and overcoming barriers to change	Introduce ideas about unhelpful thoughts, automatic thoughts and errors in thinking. To identify reasons why people stay in the pain cycle, and barriers to change. Introduce positive reframing	Cognitive behaviour change<br><br>Self-management of pain	Problem solving<br><br>Reduce negative emotions<br><br>Framing/reframing
Mindful attention control	To introduce Mindfulness as a tool to train attention and distract from pain	Principles of mind body therapies and biofeedback and visualisation	Behavioural practice<br><br>Distraction<br><br>Body changes
Balance and stretch	To promote body awareness and core strength	Guidelines on exercise<br><br>Physical therapy principles	Demonstration of behaviour<br><br>Behavioural practice
Summary of the day	To consolidate learning of the day and outline aims for final group day 3. A reminder to attend the one to one appointment with the clinical facilitator.	Acceptance and principles of self-efficacy	Action planning<br><br>Verbal persuasion about capability
I-WOTCH group based Sessions Day 3 (week 3)	Aims	Theoretical Underpinnings	Behaviour Change Taxonomy
Reflections from day two	To understand and empathise with the group and ascertain current thoughts	Social learning<br><br>Self-efficacy	Review of behaviour
Anger, irritability and frustration	Identifying reasons for negative emotions and implementing goal setting and action planning	Cognitive behaviour change<br><br>Theory of planned behaviour and reasoned action	Reduce negative emotions<br><br>Goal setting<br><br>Action planning

For peer review only - http://bmjopen.bmj.com/site/about/guidelines.xhtml

Relationships: Getting the most from your healthcare team (Part1)	To reflect on consulting behaviour and promote effective communication and constructive consultations	Biopsychosocial theory<br><br>Theory of planned behaviour and reasoned action	Information about antecedents<br>Instruction on how to perform a behaviour (communication skills)
Relationships (Part 2) Listening skills	To improve listening and communication skills	Biopsychosocial theory<br><br>Theory of planned behaviour and reasoned action	Social support (emotional)
Managing setbacks and non-drug management techniques	To know what to do when experiencing a setback or a flare up	Cognitive behaviour change<br><br>Self-efficacy	Anticipated regret<br><br>Focus on past success
Mindful distraction activity –colouring	To learn how to focus the mind away from pain thoughts and use of opioids	Principles of mind body therapies and biofeedback and visualisation	Behavioural practice<br><br>Distraction<br><br>Body changes
Stretch	To learn how to stretch muscles gently with low risk of injury and pain	Biopsychosocial theory<br><br>Self-efficacy<br>Principles of acceptance	Demonstration of behaviour<br><br>Behavioural practice
Mindfulness of Thoughts & Senses	To learn how to apply mindfulness of thoughts by detaching emotion from reality, to appreciate 'the now'	Principles of mind body therapies<br>Biofeedback and visualisation	Distraction
Summary of the day	To consolidate the days learning.	Acceptance and principles of self-efficacy	Action planning
Summary of the course	To clarify learning from past 3 group days and motivation to continue with opioid reduction	Acceptance and principles of self-efficacy	Review of behaviour<br><br>Verbal persuasion about capability
One to one session	Aim	Theoretical Underpinnings	Behaviour Change Taxonomy
Interaction one: face to face with clinical facilitator	To reflect on group learning days, agree tapering goals and generate tapering plan	Cognitive behaviour change<br><br>Motivational Interviewing	Goal setting behaviour<br><br>Action planning<br><br>Graded task<br><br>Pros and cons
Interaction two: 30 minute via telephone call with clinical	To reflect on progress and offer support during the tapering	Cognitive behaviour change	Review behaviour<br><br>Behavioural contract

For peer review only - http://bmjopen.bmj.com/site/about/guidelines.xhtml

facilitator	process	Motivational Interviewing	(adapted – as generated plan written)<br>Social reward (congratulating on effort made and progress towards tapering-verbal)
Interaction three: 30 minute via telephone with clinical facilitator	To reflect on progress and offer support during the tapering process	Cognitive behaviour change<br>Motivational Interviewing	Identification of self as role model (their own behaviour may be an example to others as they taper)
Interaction four: face to face with clinical facilitator	To reflect on progress so far and goals and discuss goals for future	Cognitive behaviour change<br><br>Motivational Interviewing	Review behaviour<br><br>Review outcome goal If applicable: discrepancy between current behaviour and goal feedback on behaviour<br><br>Goal setting (behaviour)<br><br>Goal setting (outcome)<br><br>Action planning

Feasibility Testing

Funding from the Hambelton and Richmond Clinical Commissioning Group for a community pain management service allowed us to test the feasibility of the I-WOTCH intervention. Seven people were trained by the study team to deliver the intervention (3 community team clinicians 2 nurses and 2 volunteer patients). Two courses were observed by a member of the study team to evaluate how the course content was delivered and received by both the group facilitators and the group participants (five participants in total). Discussions included, what worked well, what did not work well, and whether participants felt that the aims and objectives of the programme were met and suggestions for changes.

For peer review only - http://bmjopen.bmj.com/site/about/guidelines.xhtml

For peer review only

The second stage of feasibility was part of the pilot phase of the randomised controlled trial and involved facilitator training for the trial. Two groups were delivered in Coventry. From both stages of feasibility testing feedback was taken on board and adaptions implemented for the training (Table 3) and course content and structure (Table 4).

Table 3: Feedback and changes pilot phases I and II- Training

Feedback (Pilot phase I and II) – Training and facilitator feedback	Changes implemented
Facilitators agreed it is useful to go through the manual step by step, to gain familiarity with each component and navigate through the different stages. They preferred this rather than going through generic topics.	We incorporated this information into the training and prior to a group being delivered, if needed the study team helped to arrange meetings between the facilitators.
Facilitators felt it would be useful for all material to be emailed prior to the training to allow time for familiarisation with the manual.	Throughout the I-WOTCH study all course material was sent to facilitators prior to training.
Facilitators suggested that during the training it would be useful to actually practice some of the sessions.	Where possible during the training days we incorporated case studies and role play, as well as experiential learning of mindfulness and using the tapering app to calculate opioid reduction doses.
Facilitators suggested that it would be useful if the course slides were numbered in correspondence to the sections in the manual.	All course slides numbered and added to the manual for reference.
Facilitators also suggested that it would be useful to include the rationale for each topic into the manual, as it helped with their understanding of each topic and with their explanation to participants.	Rationale for each topic was included in the manual.

Table 4: Feedback and changes pilot phases I and II- Course content and structure

Feedback (Pilot phase I and II) participant feedback	Changes implemented
During pilot phase I, feedback favoured spreading the group sessions over three weeks (one group day per week). This was to help with consolidation of information and learning between sessions and also felt less burdensome.	In the trial the I-WOTCH group structure was delivered with this format (every Monday where possible for three weeks).
It was suggested the balance session worked well after the session on posture, to allow more understanding and connection with body.	This was changed in the I-WOTCH programme: balance and stretch was introduced on day 2 of

For peer review only - http://bmjopen.bmj.com/site/about/guidelines.xhtml

	the programme and posture and movement on day 1 of the programme.
Day 1 presented a lot of educational information on opioids and it was suggested to split this over two days to help support consolidation of understanding	The educational information was split over two days (day 1 and day 2 of the programme).
It was also suggested to move the session on pacing to after the pain cycle has been discussed, to help with the understanding of why pacing is important and can help break the unhelpful cycle.	The pain cycle was introduced and on day 1 of the programme and pacing was moved to day two of the programme.
During Pilot phase I, patients welcomed an educational DVD to help with the learning.	As part of I-WOTCH we produced an I-WOTCH education DVD which is used in the delivery of the programme, participants are able to then take this home and watch with their family and friends or keep as a resource for themselves.

Overall, the feedback regarding the content of programme was positive. Participants felt that the distraction techniques worked well and helped break up the sessions. They also valued understanding the link between mood and pain and found the case studies useful in helping to motivate them to start reducing their opioids. Facilitators and participants in both pilot phases reported that it was an informative interactive course. Observations showed good delivery and interaction between facilitators and participants, good use of questions and answer sessions and role play. Both facilitators and patients agreed it may have been more interactive had the group been larger.

Final I-WOTCH Intervention

The final I-WOTCH intervention. (Fig 3) consists of group day 1 (delivered week one), group day two (delivered week two), a one-to-one consultation with an I-WOTCH trained nurse (also in week 2 and after group day two), group day three (week three) and then two telephone consultations and a final face to face consultation to offer continual support for tapering. Each component of the intervention builds on previous knowledge and experience, and where the one-to-one consultation allows consolidation and tailoring of advice and support for tapering.

For peer review only - http://bmjopen.bmj.com/site/about/guidelines.xhtml

At the beginning of the intervention the learning is centred on pain and opioid education, with day two of the programme then introducing changes in beliefs and adapting different strategies as reduction of opioids occur. It is at this point tailoring support and motivational interviewing to action a change in beliefs is promoted through the one-to-one support sessions with an opportunity for further regulation and group cohesion/support on the wider impact of opioid reduction and long term behaviour change. The further one to ones support, self-regulation, reflection and monitoring.

One to one consultations

The one-to-one sessions with a trained I-WOTCH nurse were based on a motivational interviewing (MI) model.(27) The aims of MI are to enhance behaviour change through a patient-centred framework, where the patient is able explore personal goals, ambivalence to change and reach self-actualisation in a supportive environment. We trained the I-WOTCH nurses on the five principles of motivational interviewing: i, expressing empathy through reflective learning, ii, expressing empathy through reflective listening, iii, developing discrepancy between participant goals or values (related to opioid tapering and pain management) and their current behaviour, avoiding argument and direct confrontation, iv, adjusting to client resistance to reducing opioid reduction rather than opposing it directly and v, supporting self-efficacy and optimism. The one-to-one consultations included a review of medication, reflection on the opioid education and group session where case studies and information were presented and exploring any challenges to opioid tapering such as concerns about withdrawal. Nurses were also trained to calculate total opioid daily dose and how to use that to produce a tapering regime according to the I-WOTCH study protocol. Although MI has been widely applied in substance misuse there are limited data available for its use in opioid cessation for people with chronic non-malignant pain. A 2020 pilot study testing motivational interviewing to support opioid tapering in post joint arthroplasty surgery found a 62% increase

For peer review only - http://bmjopen.bmj.com/site/about/guidelines.xhtml

in the rate of participants returning to baseline opioid use after surgery (HR 1.62; 95%CI 1.06–2.46; p = 0.03).(28) Opioid tapering conversations maybe challenging and each participant will bring their own experiences and motivation to change, however by using MI as a tool we encouraged I-WOTCH facilitators to support participants in their tapering..(29)

One to one tapering – App

We adopted an opioid tapering regimen based on the Mayo Clinic experience as it provided some evidence to support the notion that slow tapering is unlikely to be associated with severe withdrawal symptoms and therefore likely to facilitate adherence.(30) This consisted of a 10% reduction of the original total daily dose every 7 days until a 30% of the original daily dose is reached. This is followed by a weekly decrease by 10% of the remaining dose. The 10% was rounded up to suit prescribing. For the calculation of equianalgesic doses we used the tables from the Faculty of Pain Medicine.(31) In order to ensure standardisation of tapering methodology across sites and various opioid preparations the team developed a tapering App for use by the I-WOTCH trained nurses across sites. The I-WOTCH tapering App was developed by JN and SE working with the Warwick University Clinical Trials Unit (CTU)programming team (HA, CM and AW) and provided to the nurses on a handheld tablet. The I-WOTCH tapering App was based on a mathematical algorithm applying the Mayo clinic tapering regime while accounting for UK commercial preparations. Nurses used the App to generate a participant specific tapering plan, which was synchronised to the I-WOTCH Trial database. The study team at Warwick CTU then logged into the centralised trial management website, printed and posted the tapering plan to the participant for their information and General Practitioner (GP) for prescribing.

The I-WOTCH trained nurse entered the total daily dose of the participant-specific opioid preparation into the home screen of the App (e.g. 60mg oxycodone/day). The App algorithm then calculated 10% of the total daily dose and rounded this up or down to suit prescribing. All

For peer review only - http://bmjopen.bmj.com/site/about/guidelines.xhtml

tablet, capsule or patch denominations of all opioid preparations were tabulated and added to the App to ensure the algorithm not only produced a 10% per week tapering regime but also recommended various prescribing methods (e.g., oxycodone 35 mg could be prescribed as 30 and 5mg or 20,10 and 5mg tablets or 10,10,10 and 5mg tablets).

For patch preparations we advised participants to taper using their original opioid if 10% was not achievable (e.g., 12mcg of fentanyl being the smallest step down), the app algorithm was adjusted to recommend a 20% taper at two-week intervals. Lowest dosage patch preparations were finally converted to slow release morphine equianalgesic doses and tapered accordingly.

My Opioid manager

The My Opioid Manager™ Book and App is the output of a project of Toronto Rehabilitation Institute, University Health Network. In 2010, Dr. Andrea Furlan, a Physician and Scientist at Toronto Rehabilitation Institute, developed a tool for physicians prescribing opioids for patients with chronic non-malignant pain. In 2012, the Opioid Manager™ was converted to an App for smartphones and tablets. The My Opioid Manager Book (and App) is intended to complement the Opioid Manager™ by providing the same information in a format that can be used by people with chronic pain who are on opioids, or by people who are not on opioids but who might be considering this option to help manage their chronic pain. The goal of My Opioid Manager is preparing the patient for upcoming consultations with their healthcare provider. Some of the topics discussed include: understanding the causes of various types of pains, uses of opioids and the side effects and risks, managing pain by tracking opioid trials, and tips on using opioids. For this study we Anglicised the content in terms of language used as well as name of medication brands and pictures to be more representative of the UK population.

Venue for delivering the intervention

For peer review only - http://bmjopen.bmj.com/site/about/guidelines.xhtml

Where possible the I-WOTCH intervention is delivered in the community. Factors to consider when booking a venue included, access to building, parking and public transport links, a room to accommodate participants and facilitators with chairs and equipment, stairs, lifts, kitchen facilities and room for equipment such as flipchart, laptop screen, speakers and internet access.

I-WOTCH facilitator Training

The delivery–receipt–enactment chain of the I-WOTCH intervention provided a framework for training of facilitators and defining dosage received for participants to promote behaviour change (opioid tapering).(32) The I-WOTCH training included two full days for all facilitators (clinical and lay facilitators) and an additional day for clinical facilitators only, to learn the clinical aspects of tapering, opioid specific education, generating tapering plans and motivational interviewing for the one to one consultations. The design of the training package and implementation was adapted to Kolb's experiential learning cycle (training, experience and reflective observation).(33) The training days gave all facilitators exposure to the different components of the intervention through education and use of case studies. Trainers were given copies of the I-WOTCH manual and all participant intervention materials. Throughout the training days facilitators had the opportunity to ask questions and get clarity on any of the topics being covered. At the end of the training a short assessment was completed by each facilitator. If any of the facilitators scored below 70% they were then contacted by phone to go over any areas needing further explanation and offered further training if needed.

Discussion

We have used a methodological approach to developing an intervention for opioid reduction for people with chronic non-malignant pain. Based on the COPERS intervention for the management of pain, best available empirical evidence at the time, and consultation with lay people we have developed a manualised intervention and training package. It has been piloted, revised and adapted considering all feedback received. The I-WOTCH intervention has the

For peer review only - http://bmjopen.bmj.com/site/about/guidelines.xhtml

potential to help people reduce their opioid use and improve their overall quality of life. We are not aware of any other programme of analogous interventions targeting similar populations. Previous non-pharmacological interventions have included mindfulness, cognitive behaviour therapy and meditation and the use of electro acupuncture which showed no reduction in the number of participants who ceased their opioid use.(4) The I-WOTCH intervention differs in that it combines group and one to one support, with the mechanisms of change and opioid reduction targeted through peer support, education, case studies, reflection and motivational interviewing. It is a time and resource intensive intervention, however, having a multi component intervention will increase the potential to address the complex psychological, social and physical aspects of opioid tapering. We have developed an opioid tapering App which can be used to calculate individual opioid tapering plans.

The roll out and scalability of the I-WOTCH training has been considered, a step by step manual with materials to set up and deliver the programme was created. The I-WOTCH facilitator training can be delivered to groups of clinicians and ongoing support given through-out the delivery of the intervention. The I-WOTCH trial will allow us to assess: the delivery of the intervention on a large scale, the training of multiple facilitators and managing the group element of the programme.

Conclusion

We have designed an opioid reduction intervention package suitable for testing in a randomised controlled trial.

Collaborators I-WOTCH team:

Sharisse Alleyne, University of Warwick, Charles Abraham, Deakin University, Shyam Balasubramanian, Department of Anaesthesia & Pain Medicine University Hospitals Coventry and Warwickshire NHS Trust, Lauren Betteley, University of Warwick, Katie Booth,

For peer review only - http://bmjopen.bmj.com/site/about/guidelines.xhtml

University of Warwick, Kirstie Haywood, University of Warwick, Sheeja Manchira Krishnan, The University of York, Ranjit Lall, University of Warwick. Andrea Manca, The University of York. Dipesh Mistry, University of Warwick, Vivien P Nichols, University of Warwick, Emma Padfield, University of Warwick, Anisur Rahman, Kate Seers, University of Warwick, University College London, Cynthia Paola Iglesis Urrutia,.

Contributors

All authors read and approved the manuscript. HS and SE are Co-Chief Investigators and oversee the running of the study. All named authors contributed to the design and/or delivery of the I-WOTCH intervention. HS, SE, JS and DC were involved in the design of the I-WOTCH intervention and design and delivery of facilitator training. CBT contributed to the design and delivery of the I-WOTCH intervention, providing feedback on all materials and a trained facilitator. ADF developed My Opioid Manager, the content was anglicised for this study and also contributed to the design of the overall I-WOTCH intervention. SE, JN HA, CM and AW developed the I-WOTCH Opioid tapering App. MU, NYKT and SJCT contributed to the design of the intervention, training manuals and to this manuscript.

Ethical Approval

Ethics approval was given by Yorkshire & The Humber - South Yorkshire Research Ethics Committee on September 13th, 2016 **16/YH/0325**.

Acknowledgments

We would like to thank Ms Sally Brown for her valuable input in the development of the intervention. We would also like to thank all of our PPI volunteers and the Clinical Research Network (North East and Cumbria) for hosting the events which provided valuable insight to the structure and content of the intervention. We would like to thank our clinical and lay facilitators who attended training, delivered the intervention and provided valuable feedback. We would like to thank all participants involved in the pilot stages of the intervention. We

For peer review only - http://bmjopen.bmj.com/site/about/guidelines.xhtml

would also like to thank Dr Alison Hipwell for her contribution to the intervention development and Dr Celia Bernstein and Dr Jodie Westhead for their contribution to the I-WOTCH study.

Figure 1: Stages of I-WOTCH intervention development

Figure 2: Reducing opioids for people with chronic non-malignant pain

Figure 3: Final model of I-WOTCH intervention

Data Sharing statement

No additional data available

References

1. Vos T, Abajobir AA, Abate KH, Abbafati C, Abbas KM, Abd-Allah F, Abdulkader RS, Abdulle AM, Abebo TA, Abera SF, Aboyans V. Global, regional, and national incidence, prevalence, and years lived with disability for 328 diseases and injuries for 195 countries, 1990–2016: a systematic analysis for the Global Burden of Disease Study 2016. The Lancet. 2017 Sep 16;390(10100):1211-59.

2. Bridges S. Health Survey for England 2011, Volume 1, Health , social care and lifestyles. HSCIC; 2012. p. 291-306.

3. International Association for the Study of Pain. Chronic Pain has arrived in the ICD-11 [Internet]. Washington: International Association for the study of Pain: 2019 Jan 17 [cited 2021 April 30]. Available from https://www.iasp pain.org/PublicationsNews/NewsDetail.aspx?ItemNumber=8340.

For peer review only - http://bmjopen.bmj.com/site/about/guidelines.xhtml

For peer review only

4. Mathieson S, Maher CG, Ferreira GE, Hamilton M, Jansen J, McLachlan AJ, Underwood M, Lin CW. Deprescribing Opioids in Chronic Non-cancer Pain: Systematic Review of Randomised Trials. Drugs. 2020 Jul 31:1-4.

5. Openprecribing, [Cited May 2021] Abailable from https://openprescribing.net/analyse/#org=stp&numIds=4.7.2&denom=nothing&selectedTab=chart

6. Baldini A, Von Korff M, Lin EH. A review of potential adverse effects of long-term opioid therapy: a practitioner's guide. The primary care companion to CNS disorders. 2012;14(3).).

7. Chou R, Turner JA, Devine EB, Hansen RN, Sullivan SD, Blazina I, Dana T, Bougatsos C, Deyo RA Ann Intern Med. 2015 Feb 17;162(4):276-86. doi: 10.7326/M14-2559. Review. PMID:25581257

8. Kurita GP, Højsted J, Sjøgren P. Tapering off long-term opioid therapy in chronic non-cancer pain patients: a randomized clinical trial. Eur J Pain. 2018;22(8):1528-1543

9. Covington EC, Argoff CE, Ballantyne JC, Cowan P, Gazelka HM, Hooten WM, Kertesz SG, Manhapra A, Murphy JL, Stanos SP Jr, Sullivan MD. Ensuring Patient Protections When Tapering Opioids: Consensus Panel Recommendations. Mayo Clin Proc. 2020 Oct;95(10):2155-2171. doi: 10.1016/j.mayocp.2020.04.025. PMID: 33012347.

10. Sandhu H, Underwood M, Furlan AD, Noyes J, Eldabe S. What interventions are effective to taper opioids in patients with chronic pain?. bmj. 2018 Sep 27;362.

11. O'Cathain A, Croot L, Sworn K, Duncan E, Rousseau N, Turner K, Yardley L, Hoddinott P. Taxonomy of approaches to developing interventions to improve health: a systematic methods overview. Pilot and feasibility studies. 2019 Dec;5(1):1-27.).

For peer review only - http://bmjopen.bmj.com/site/about/guidelines.xhtml

12. Craig P, Dieppe P, Macintyre S, Michie S, Nazareth I, Petticrew M. Developing and evaluating complex interventions: the new Medical Research Council guidance. BMJ (Clinical research ed). 2008;337:a1655.]

13. Carnes D, Homer K, Underwood M, Pincus T, Rahman A, Taylor S.J.C. Pain management for chronic musculoskeletal conditions: the development of an evidence based and theory informed pain self-management course. BMJ Open. 2013; 3:e003534.doi:10.1136

14. Univeristy of Warwick. UNTRAP Leaflet [Internet]. Coventry: Univeristy of Warwick [Cited May 2021]. Available from https://warwick.ac.uk/fac/cross_fac/healthatwarwick/untrap/untrapleaflet.pdf.

15. Turk DC, Wilson H, Swanson KS, Ebert M, Kerns R. The biopsychosocial model of pain and pain management. Cambridge: Cambridge University Press; 2011.

16. Abraham C, Michie S. A taxonomy of behavior change techniques used in interventions. Health Psychol 2008;27:379–87

17. Richards, Georgia C.a,*; Lluka, Lesley J.b; Smith, Maree T.c; Haslam, Catherined; Moore, Brendanc,e; O'Callaghan, Jamese; Strong, Jennyf Effects of long-term opioid analgesics on cognitive performance and plasma cytokine concentrations in patients with chronic low back pain: a cross-sectional pilot study, PAIN Reports: July/August 2018

18. Diamond A. Executive functions. Annual review of psychology. 2013 Jan 3;64:135-68.

19. Bandura A. Self-efficacy: toward a unifying theory of behavioral change. Psychological review. 1977 Mar;84(2):191.

For peer review only

20. Ajzen I, Fishbein M. Understanding attitudes and predicting social behavior. London: Pearson Education, 1980

21. Ajzen I, Fishbein M. Belief, attitude, intention and behaviour: an introduction to theory and research. Higher Education; 1976.

22. Bandura A. Social foundations of thought and action. Englewood Cliffs, NJ. 1986;1986(23-28).

23. Morley S, Shapiro DA, Biggs J. Developing a treatment manual for attention management in chronic pain. Cogn Behav Ther 2004;33:1–11.

24. Kuhl J, Beckmann J, editors. Action control: From cognition to behavior. Springer Science & Business Media; 2012 Dec 6.

25. Hayes SC, Strosahl KD, Wilson KG. Acceptance and commitment therapy: an experiential approach to behavior change. New York: Guilford Press, 2004.

26. Carnes D, Homer K, Underwood M, Pincus T, Rahman A, Taylor SJ. Pain management for chronic musculoskeletal conditions: the development of an evidence-based and theory-informed pain self-management course. BMJ open. 2013 Nov 1;3(11).

27. Miller WR, Rollnick S. Motivational interviewing: Preparing to change addictive behaviour. New York: Guilford. 1991.

For peer review only

28. Hah JM, Trafton JA, Narasimhan B, Krishnamurthy P, Hilmoe H, Sharifzadeh Y, Huddleston JI, Amanatullah D, Maloney WJ, Goodman S, Carroll I. Efficacy of motivational-interviewing and guided opioid tapering support for patients undergoing orthopedic surgery (MI-Opioid Taper): A prospective, assessor-blind, randomized controlled pilot trial. EClinicalMedicine. 2020 Nov 1;28:100596.).

29. Crawley A, Murphy L, Regier L, McKee N. Tapering opioids using motivational interviewing. Canadian Family Physician. 2018 Aug 1;64(8):584-7.

30. Berna C, Kulich RJ, Rathmell JP. Tapering long-term opioid therapy in chronic noncancer pain: evidence and recommendations for everyday practice. InMayo Clinic Proceedings 2015 Jun 1 (Vol. 90, No. 6, pp. 828-842). Elsevier.

31. Faculty of Pain Medicine of Royal College of Anaesthetists. Dose equivalents and changing opioids [Internet]. London: Faculty of Pain Medicine of Royal College of Anaesthetists. Available from: https://fpm.ac.uk/opioids-aware-structured-approach-opioid-prescribing/dose-equivalents-and-changing-opioids

32. Sandhu HK, Abraham C, Alleyne S, Balasubramanian S, Betteley L, Booth K, Carnes D, Furlan AD, Haywood K, Urrutia CP, Lall R. Testing a support programme for opioid reduction for people with chronic non-malignant pain: the I-WOTCH randomised controlled trial protocol. BMJ open. 2019 Aug 1;9(8):e028937.

33. Kolb DA. Experience as the source of learning and development. Upper Sadle River: Prentice Hall. 1984.

For peer review only - http://bmjopen.bmj.com/site/about/guidelines.xhtml

For peer review only

For peer review only - http://bmjopen.bmj.com/site/about/guidelines.xhtml

Figure 1: Stages of I-WOTCH intervention development

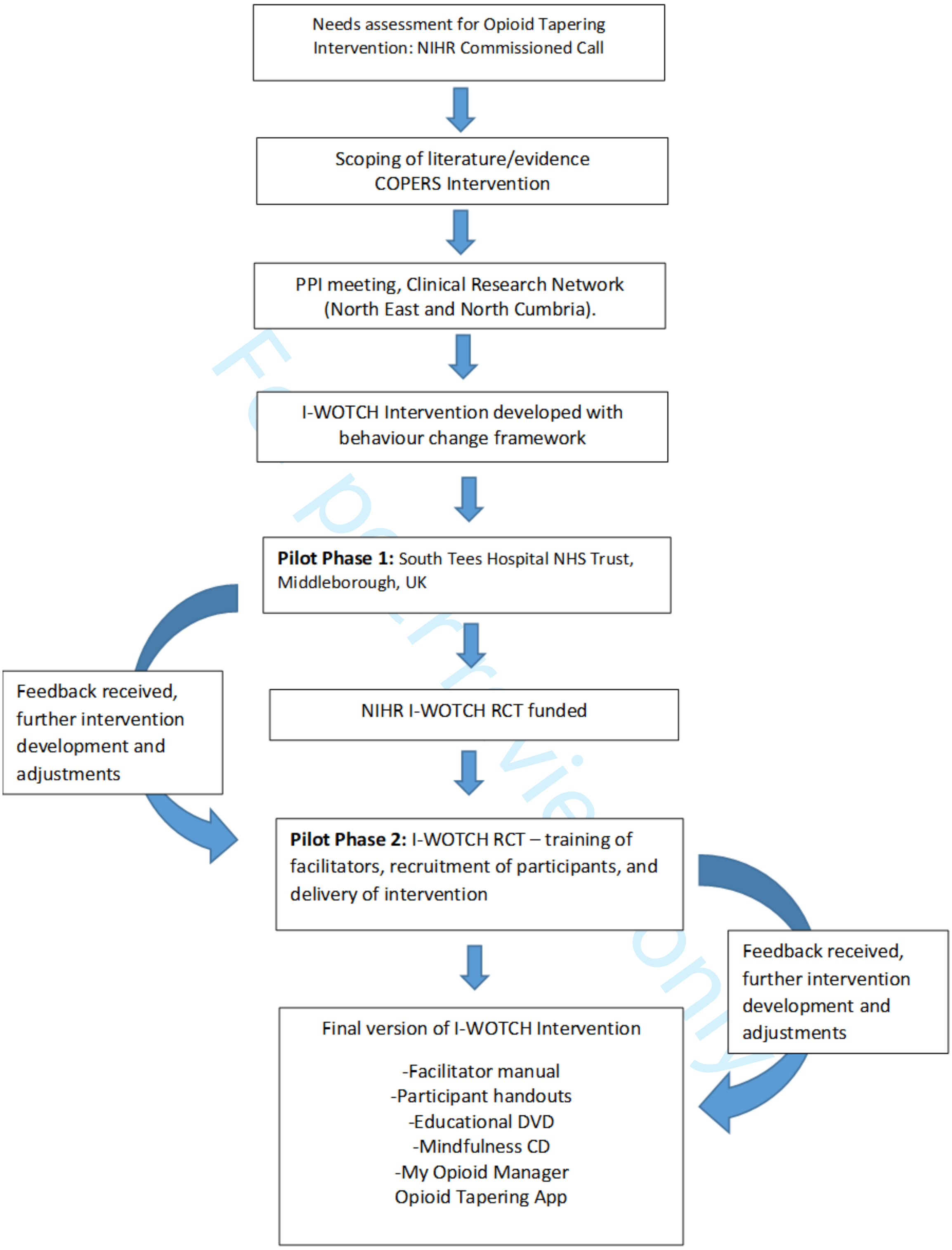

Legend:
COPERS - coping with persistent pain, effectiveness research into self-management (13)
I-WOTCH- Improving the Wellbeing of Opioid Treated Chronic Pain
NIHR – National Institute of Health Research
PPI - Patient and Public Involvement
RCT – Randomised Controlled Trial

Figure 2: Reducing Opioids for peoplewith chronic non malignant pain

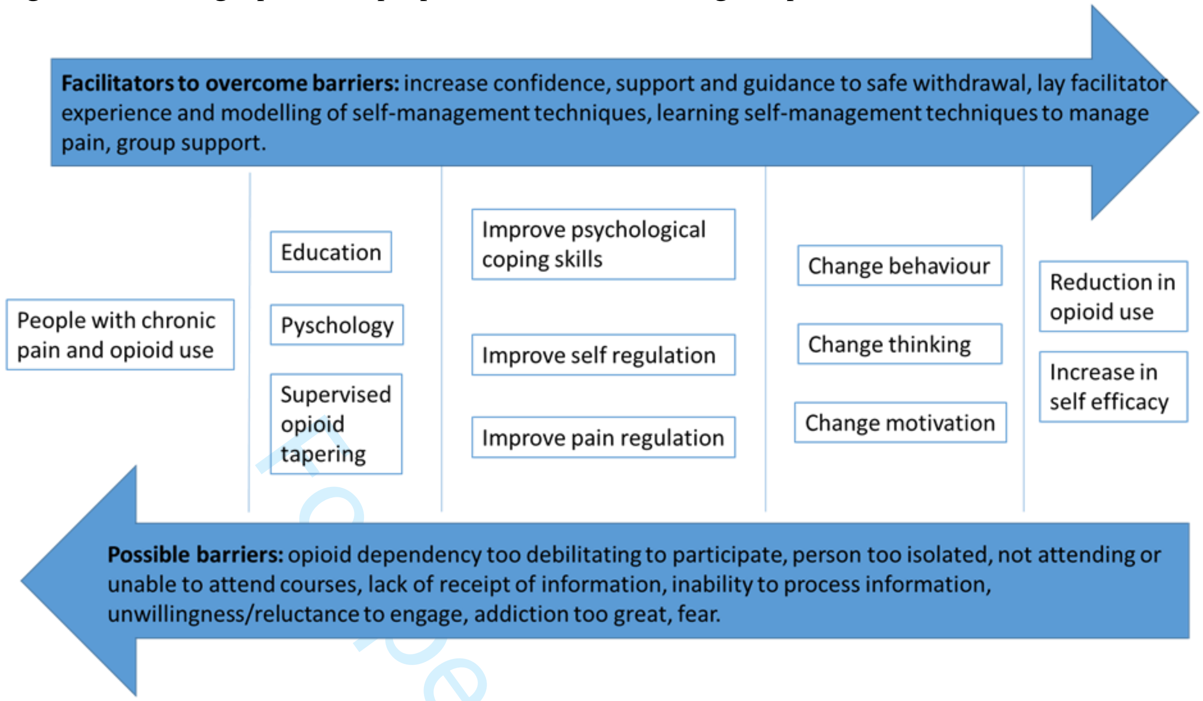

For peer review only

Figure 3: Final model of I-WOTCH intervention

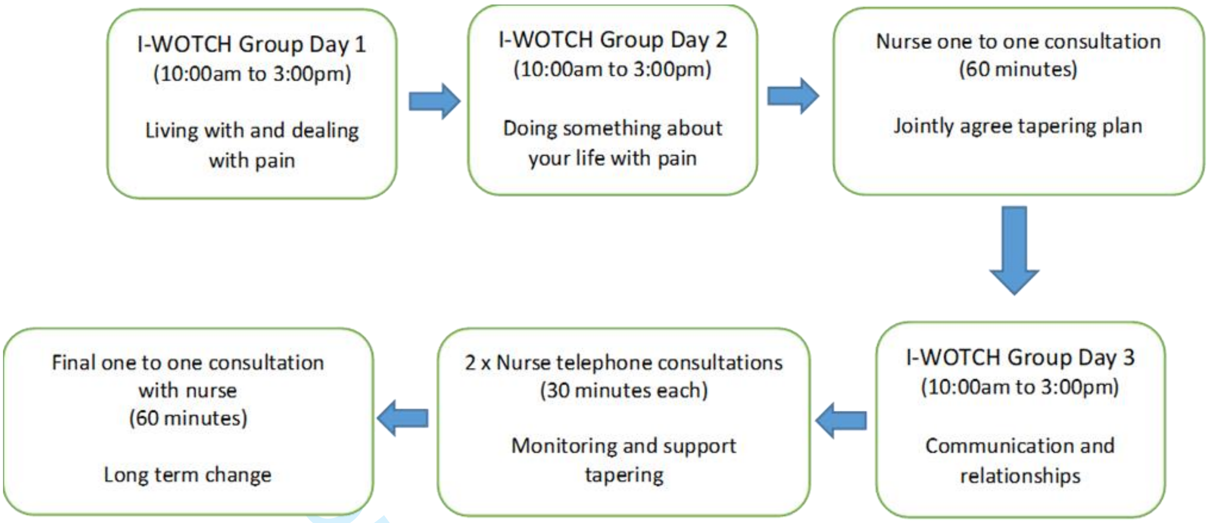

For peer review only
